# Obsessive compulsive symptom dimensions are linked to altered white-matter microstructure in a community sample of youth

**DOI:** 10.1038/s41398-022-02013-w

**Published:** 2022-08-10

**Authors:** Rachael G. Grazioplene, Colin G. DeYoung, Michelle Hampson, Alan Anticevic, Christopher Pittenger

**Affiliations:** 1 Yale University, Department of Psychiatry, New Haven, CT USA; 2 University of Minnesota, Department of Psychology, Minneapolis, MN USA; 3 Yale University, Department of Radiology and Biomedical Imaging, New Haven, CT USA

**Keywords:** Psychiatric disorders, Diagnostic markers

## Abstract

Obsessive-compulsive symptoms (OCS) are common in school-aged children and predict the development of obsessive compulsive disorder (OCD). White-matter abnormalities have been described in OCD, but the white matter correlates of OCS in the developing brain are unclear. Some correlates of OCS (or a diagnosis of OCD) may reflect correlates of a transdiagnostic or even general psychopathology factor. We examined these questions in a large sample of typically developing youth (*N* = 1208), using a hierarchical analysis of fixel-based white matter measures in relation to OCS and general psychopathology. General psychopathology was associated with abnormalities in the posterior corpus callosum and forceps major in an age-dependent manner, suggesting altered maturation (specifically, hypermaturation in younger subjects). A unidimensional measure of OCS did not associate with any white-matter abnormalities, but analysis of separate OCS dimensions (derived from factor analysis within this sample) revealed the ‘Bad Thoughts’ dimension to associate with white-matter abnormalities in dorsal parietal white-matter and descending corticospinal tracts, and the ‘Symmetry’ dimension to associate with abnormalities in the anterior corpus callosum. Repetition/checking and Symmetry OCS were additionally associated with posterior abnormalities overlapping with the correlates of general psychopathology. Contamination symptoms had no white-matter correlates. Secondary analysis of fractional anisotropy (FA) revealed distinct white-matter abnormalities, suggesting that fixel-based and FA analyses identify distinct features of white matter relevant to psychopathology. These findings suggest that OCS dimensions correlate with dissociable abnormalities in white matter, implicating separable networks. Future studies should examine these white-matter signatures in a longitudinal framework.

## Introduction

Obsessive Compulsive Disorder (OCD) is a common, often debilitating mental illness that imposes substantial personal and societal burden. Clinical OCD affects up to 2.7% of the population; another 6–10% of individuals older than 13 exhibit subclinical obsessive compulsive symptoms (OCS) [1]. OCS include obsessions (repetitive urges, images, or thoughts) as well as compulsions (repetitive behaviors, such as checking or praying). Population-based studies demonstrate a continuum between subclinical OCS and clinically severe OCD in school-aged samples [2–4]. OCD and OCS are markedly heterogeneous; factor analytic studies suggest several dissociable dimensions, including preoccupations with symmetry, contamination, and intrusive ‘bad thoughts’ [3, 5]. OCD and OCS are also characterized by significant comorbidity; up to 90% of individuals with a diagnosis of OCD have at least one comorbid DSM diagnosis, with mood and anxiety disorders being the most common [6]. Indeed, there is emerging evidence for a general psychopathology factor (sometimes called the p-factor), which may explain a significant fraction of symptom variance and functional impairment across major neuropsychiatric diagnoses [7–9]. Studies of the pathophysiology of OCS and OCD across development must contend with these complexities.

OCD is associated with a range of functional and structural brain abnormalities that have slowly come into focus [10–17]. Studies of brain white matter, in particular, have advanced rapidly in recent years [13, 18, 19]. Here, too, questions arise as to whether white-matter abnormalities are specific to OCS/OCD or are rather a reflection of general psychopathology [7–9].

Studies of adult OCD have reported a “deficit” pattern of white matter abnormalities, particularly in the cingulum bundles [10, 20], corpus callosum [21, 22], orbitofrontal cortex [21], and the anterior limb of the internal capsule [22]. A large recent study from the ENIGMA collaborative identified white-matter microstructural abnormalities in the posterior thalamic radiation in adult OCD, focusing attention on posterior cortical regions that have been relatively neglected in the OCD literature to date [13]. Fewer studies have explored OCD and OCS in children and adolescents; the emerging pattern is of positive associations between white-matter coherence and OC symptom presence/severity [22], although the recent ENIGMA study found no white-matter abnormalities in a pediatric OCD subsample [13]). The largest study examining white matter correlates of pediatric OC-spectrum pathology identified a novel association between symptom severity and fractional anisotropy in a parietal subsection of corticostriatal white-matter radiations [23]. Sample sizes examined in most previous DWI-based OCD studies have been modest; variability may derive from sample heterogeneity, psychiatric comorbidities, method variance, and illness chronicity. No published studies have examined the white matter correlates of distinct OCS dimensions.

Here, we examine the white matter correlates of OCS in a large sample of youth, ages 8–21, from the Philadelphia Neurodevelopmental Cohort (PNC) [24]. This is a key age range for the development of OCS and OCD [25]. We use a hierarchical approach to distinguish white matter correlates of OCS from those of general psychopathology. We characterize the white matter correlates both of overall OCS and of specific OCS subtypes, using symptom dimensions derived using factor analysis of the same sample [3]. We employ MRtrix3’s fixel-based diffusion imaging pipeline, which has not previously been used to analyze white matter correlates of OCS. This recently developed DWI analysis approach [26, 27] represents an advance on traditional tensor model-derived diffusion metrics in that it estimates white-matter properties for individual fiber bundle populations within voxels that contain complex crossing architecture; fixel-based analysis has been shown to better capture anatomically-established ground truth than tensor-based analysis [28]. Our primary focus here is on the fixel-based analyses, which have the potential to allow greater insight into the nature of white-matter abnormalities than fractional anisotropy (FA), an older measure. We additionally include analyses using FA, to better compare with the older literature using this measure. This combination of innovations in a large developmental sample creates the opportunity for new insight into the structural correlates of OCS in childhood.

## Methods

### Sample characteristics

Data were drawn from the Philadelphia Neurodevelopmental Cohort, which has been described previously [29]. The institutional review boards of the Children’s Hospital of Philadelphia and the University of Pennsylvania approved all study procedures. Briefly, participants were recruited by the Center for Applied Genomics at the Children’s Hospital of Philadelphia through a healthcare network of more than 30 clinics in and around the Philadelphia greater metro area (Pennsylvania, Delaware, and New Jersey). Patients who entered these community clinics between 2006–2012 were approached for participation. Following informed consent (or parental consent and patient assent) to be contacted for future participation, patients were screened for eligibility based on age (8–21 years), English proficiency, and absence of significant developmental delays or major physical disability. 9498 participants were enrolled and completed clinical assessments. A random subsample (*N* = 1601) underwent multimodal MRI imaging, including diffusion-weighted imaging (DWI). Of these 1601, 1312 had complete OCS scores and complete DWI datasets. 1208 datasets remained following conservative quality control. Details of the QC and data exclusion procedures can be found in the supplementary methods.

In the PNC data, psychopathology, trauma history, and cognitive measures all differ between racial groups [30], presumably because race serves as a marker for socioeconomic and environmental variance and chronic stress; we therefore included self-reported race (White, Black, and Other; dummy coded) as a covariate in all analyses. Age, biological sex, and cognitive ability have all been previously linked to white-matter microstructure and are associated with psychopathology risk [31]. We therefore included these variables as covariates in all analyses.

### Clinical assessment

Psychopathology was assessed using the GOASSESS, a computerized structured interview version of the K-SADS that is designed to capture a broad range of psychopathology across adolescence and young adulthood, described in detail elsewhere [29, 32, 33]. Briefly, the GOASSESS determines lifetime incidence of symptoms from major domains of psychopathology, including mood disorders (major depression, mania), behavioral problems (conduct disorder, oppositional defiant disorder), eating disorders (bulimia, anorexia), anxiety (generalized anxiety disorder, social phobia, specific phobia, agoraphobia, post-traumatic stress disorder, obsessive compulsive disorder, panic disorder, separation anxiety), ADHD, and suicidal thinking and behavior. Two additional screening tools are embedded in the GOASSESS to better capture psychosis spectrum symptoms: positive symptoms are measured using the PRIME Screen-Revised (PS-R [32]), and negative/disorganized symptoms are assessed using the Scale of Prodromal Symptoms (SOPS; [1]). Apart from the PS-R, all items are dichotomous; Likert-style ratings from the PS-R were trichotomized for use in the present analyses (ratings of 0 → 0, 1–2 → 1, 3–7 → 2).

### Cognition

Cognitive ability was assessed using the Penn Computerized Neurocognitive Battery (CNB). Full details of the battery are reported elsewhere [34]. Briefly, the CNB includes 12 subtests that span domains of executive and complex cognition, social cognition, and episodic memory.

### Trauma History

Traumatic Stressful Events (TSEs) are measured as part of GOASSESS. Lifetime exposure to eight different traumatic event types was queried: being attacked or badly beaten; any experience of sexual abuse; being threatened with a weapon; being worried that someone close would be killed or hurt badly; seeing or hearing someone hurt badly or killed; being threatened with a weapon; being upset by seeing a dead body; and being in a bad accident. Trauma was indexed in terms of summed Traumatic Stressful Events.

### Factor score derivation: general cognitive ability, general psychopathology and OCS domains

The factor structure of Obsessive-Compulsive Symptoms (OCS) has been previously reported in this sample [3]. Barzilay et al. [3] determined that the best fit model comprised four OCS factors: Bad Thoughts, Symmetry, Repetition/Checking, and Contamination. We rederived this factor structure for our analysis, using the 16 items from the GOASSESS that measured symptoms of OCD. Following [3], we excluded the one item that measured hoarding. The factor model was fit based on tetrachoric correlations among OCS items using the fa function of the “psych” package in R [35] employing oblimin rotation and using the tenBerge option (to preserve the oblique rotation) to estimate factor scores. Given the relatively limited number of OCS items, we chose not to impute missing data for the OCS model, retaining factor scores only for those individuals with complete data for the 16 OCS items (*N* = 8898 of 9496).

To compute general psychopathology factor scores for use in regression analyses, we used the 97 non-OCD-related GOASSESS items to derive a single-factor model, using a mix of tetrachoric and polychoric correlations (cor = “mixed”) to accommodate the trichotomized PRIME items together with the dichotomous GOASSESS items.

We used accuracy scores from all twelve subtests of the CNB to estimate a cognitive ability factor, or “*g*” factor, for use as a covariate in imaging analyses. Fit statistics indicated that a single-factor solution captured 29% of the variance in accuracy scores, with loadings ranging from 0.42 to 0.78. Factor scores from this single-factor solution were estimated and stored as a measure of “g” for each participant.

### DWI acquisition and preprocessing using MRtrix3

Diffusion-Weighted Imaging (DWI) scans were collected between 2009 and 2013 on the same 3T Siemens TrioTim scanner and used the same head coil (32 channel) and the same acquisition protocol. A pair of DWI scans were obtained using a twice-refocused spin-echo SS EPI sequence, with a total of 64 directions with *b* = 1000 and 7 unweighted (*b* = 0) scans; voxel size 1.875 × 1.875 × 1.875, PE direction = AP, TR/TE = 8100/81. Full acquisition details are described in [26, 36].

All participants’ data were subjected to visual inspection by an experienced analyst (RGG) prior to inclusion in the analysis pipeline; 90 participants were excluded based on the presence of visually apparent MR artifacts (venetian blinding artifacts, FOV errors, and cerebellar hyperintensities). Fixel-based processing steps were conducted using MRtrix3 software suite (version RC3), according to the procedures outlined in the MRtrix3 documentation [26]). Full details of the analytic workflow can be found in Supplementary Materials. Briefly, images were denoised, eddy corrected, and coregistered to a common, sample-specific template. Fixel-based analysis generates three measures of white-matter integrity: fiber density (FD), fiber bundle cross-section (FC), and combined fiber density and cross-section (FDC; [26]). FD captures the volume of the intra-axonal restricted compartment, while FC is a more macrostructural approximation of the relative white-matter bundle size (relative to the study population); FDC is simply, a combined measure that modulates density by cross-section [27]. Mean framewise displacement and contrast-to-noise (CNR) were extracted, per-subject, and exported for use as covariates. Factor analysis was used to compute the shared variance between these two indices of scan quality, and these derived factor scores are referred to as “QCmetric” in analytic models.

### ROI-based analyses

We examined 14 regions of interest (ROIs), identified a priori based on previous literature on OCD. ROIs were: right/left OFC pathway, right/left cingulum bundle, right/left uncinate fasciculus, right/left posterior thalamic radiation, right/left sagittal stratum, left superior corticostriatal tract, and genu, body, and splenium of the corpus callosum (see Fig. S1).

Mean FD, FC, and FDC in each ROI were extracted for each participant and stored for external analysis in R [37]. We first examined the association between the single-factor measure of OCS and each ROI, covarying for p-factor score, sex, age, race, *g*, and scan quality. P-values were penalized using FDR correction. We then conducted multiple regression analyses to test for associations between ROI-based FD/FC/FDC and each of the four OCS dimensions. Finally, we used interaction models to test whether any associations between fixel-based metrics and OCD traits differed as a function of age.

To examine FA associations in these same ROIs, fixel-based ROI masks were transformed into voxel-based binarized masks at the population template level, and FA values were extracted for all subjects for each of the 14 masks for use in multiple regression analysis. Regression models were run as on fixel data.

### Whole-brain analyses

Whole-brain tractography was performed using a sample-specific template, and the resulting 20-million streamline tractogram was reduced using SIFT [38] to contain 2 million streamlines. Whole-brain statistical testing using connectivity-based fixel enhancement was performed with the fixelcfestats command. This technique employs the GLM with non-parametric permutation testing (5000 permutations) and performs threshold-free cluster enhancement based on underlying tractography.

Given the large age range of this sample and the drastic white-matter maturation that occurs during adolescence, we first explored whether there was a significant interaction between age and any of the symptom dimensions (General psychopathology, OCS severity, Bad Thoughts, Need for Symmetry, Repetition/Checking, and Contamination) when predicting fixel-based metrics at the whole-brain level. We then investigated the main effects of each OCS dimension on the white matter at the whole-brain level, given that age-by-trait interactions and direct effects may not be colocalized. For any results that survived stringent significance testing, we performed tractography using the significant ROIs as probabilistic tractography seeds to visualize which fiber pathways the significant results were most likely to belong to.

To test the same set of whole-brain associations with FA, the fixel mask was transformed into a binarized voxel mask (with no additional thresholding), and the MRtrix3 command “mrclusterstats” was used to explore whole-brain associations between voxel-wise FA and the target psychopathology dimensions. All models employing mrclusterstats were run with 5000 permutations and employed threshold-free cluster enhancement.

Given the substantial trauma history in this sample and a previous report that trauma was linked to OCS in this sample [39], we conducted a post-hoc analysis with numbers of Traumatic Stressful Events as a covariate.

#### Nonlinear age effects

In response to suggestions that arose during the peer review process, we conducted an additional set of exploratory analyses to examine nonlinear age effects in our central models of interest [40]. First, we added a squared age term to the models that evaluated direct associations between traits and white-matter metrics. We also ran six additional interaction models that included a squared age interaction term (age^2^ × p-factor, age^2^ × Bad Thoughts, age^2^ × Repetition/Checking, age^2^ × Symmetry, age^2^ × Contamination, age^2^ × OCS-factor; see Supplementary Table 7).

#### Age and cognition (*g*-factor) effects

Since our sample spanned a period of accelerated white-matter development and our models included several age-interaction effects, we conducted a supplemental evaluation of key contrasts to summarize age effects for all white-matter metrics. These included contrasts for both linear and nonlinear effects of age (Supplementary Table 5). In addition, we include supplemental analyses that summarize associations between cognition (g-factor scores) and all white-matter metrics, including linear and nonlinear interactions with age (Supplementary Table 6).

## Results

### Sample characteristics

Demographic characteristics for the final imaging analytic subset are shown in Table 1, including summary statistics for symptom scores.

**Table 1:** Sample characteristics.

Variable	
*Sex*	
%Male	47.0%
%Female	53.0%
Mean age (SD)(range)	14.19 (3.33) (8–21)
Maternal education in years (SD) (range)	14.28 (2.43) (8–20)
*Ethnicity*	
% White	46.50%
% Black	43.13%
% Other	10.37%

Factor analysis of OCS identified a 4-factor solution like that described previously in this sample [39]. The four factors corresponded to the canonical OCD symptom dimensions of ‘Bad Thoughts’, ‘Symmetry’, ‘Repetition/Checking’, and ‘Contamination’ [5]. Model summary statistics indicated that this four-factor model was a good fit, with four factors explaining 57% of the item variance and primary factor item loadings between 0.55 and 0.90 for each factor (see Supplementary table S2A for EFA results).

The single-factor OCS model captured 55% of the variance in symptom endorsement, with factor loadings ranging from 0.66 to 0.78.

**Fig. 1:**
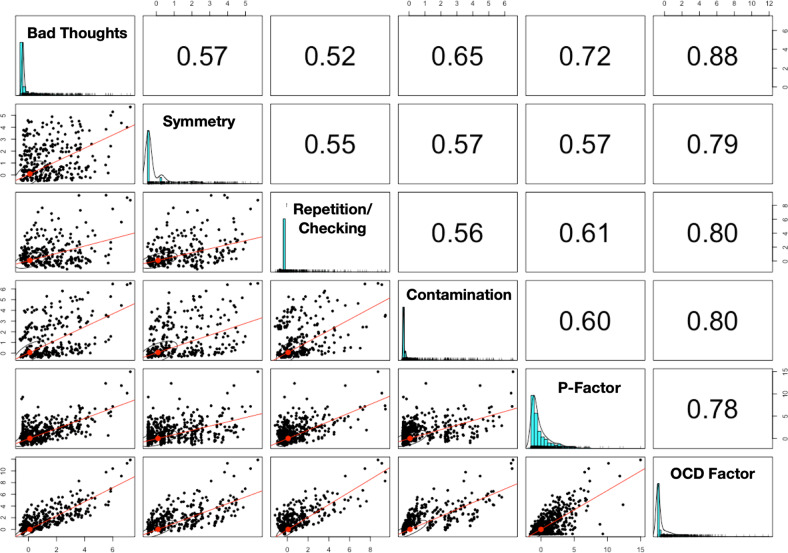
Correlations among extracted factor scores in the imaging subset. The diagonal contains frequency histograms for each trait. The upper diagonal contains zero-order correlations, and the lower diagonal presents scatterplots of these correlations.

We additionally generated a ‘General psychopathology’ factor, or p-factor. The general psychopathology factor (derived from the other 97 GOASSESS items) captured 30% of the variance in item endorsement; factor loadings ranged from 0.23 to 0.71, with the majority (between 1st and 3rd quartiles) falling between 0.48 and 0.62. Factor score distributions and zero-order correlations are presented in Fig. 1.

### Region of interest fixel analyses

Our planned analysis was based on ROIs derived from previous studies using FA analysis of white matter in OCD and OCS. Fourteen white-matter ROIs were defined based on existing literature (see Methods; Fig. S1). We performed a series of analyses examining FD, FC, and FDC in these ROIs in conjunction with general psychopathology, age, gender, OCD overall symptom severity, and the four OCD symptom dimensions. Uncorrected *p*-values for the associations between OCS dimensions and FD, FC, and FDC in these ROIs ranged from 0.005 to 1.00; none of these associations were significant following FDR correction for multiple testing (Supplementary Table 3).

### Whole-brain fixel analyses

**Fig. 2:**
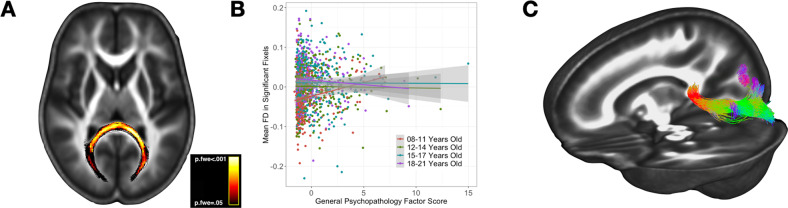
Fixel correlates of general psychopathology. There was an age-dependent association between p-factor scores and FD in the splenium of the corpus callosum. Panel (A) shows the significant effect mapped on an axial plane, with the heatmap representing family-wise error corrected p-values ranging from 0.05 to 0.007 (brighter values are more significant). Panel (B) demonstrates the post-hoc visualization of the interaction effect, with age ranges defined by quartiles. Panel (C) displays a tractogram to further characterize the anatomy of the results; the significant ROI was used as a seed region and probabilistic tractography was used to construct the tractogram.

We next performed whole-brain exploratory analyses, controlling for multiple comparisons, to identify brain-wide significant correlates of psychopathology. First, we examined associations of fiber density (FD) with general psychopathology, covarying for cognitive function (g factor), age, race, and gender (see models below, Box 1). There were no significant main effects of general psychopathology, but there was a significant interaction between age and the general psychopathology score on FD in a large region in the splenium of the corpus callosum (*d* = −0.022, p.FWE = 0.03; Fig. 2A). Post-hoc quantile-based visualization of this effect demonstrated a positive association between FD and age in the younger age range (8–11 years), with a trend toward an inverse association in the highest age range (18–21 years; Fig. 2B. Visualization of streamlines originating in the region of significant effect extended through the forceps major into the posterior parietal and occipital lobes (Fig. 2C).

We next tested for correlates of aggregate OCS severity (see Box 2). No effects of OCS severity, or age on FD, FC, or FDC survived FWE correction for brain-wide significance. Incorporation of an OCS × age interaction term similarly produced no significant effects.

**Fig. 3:**
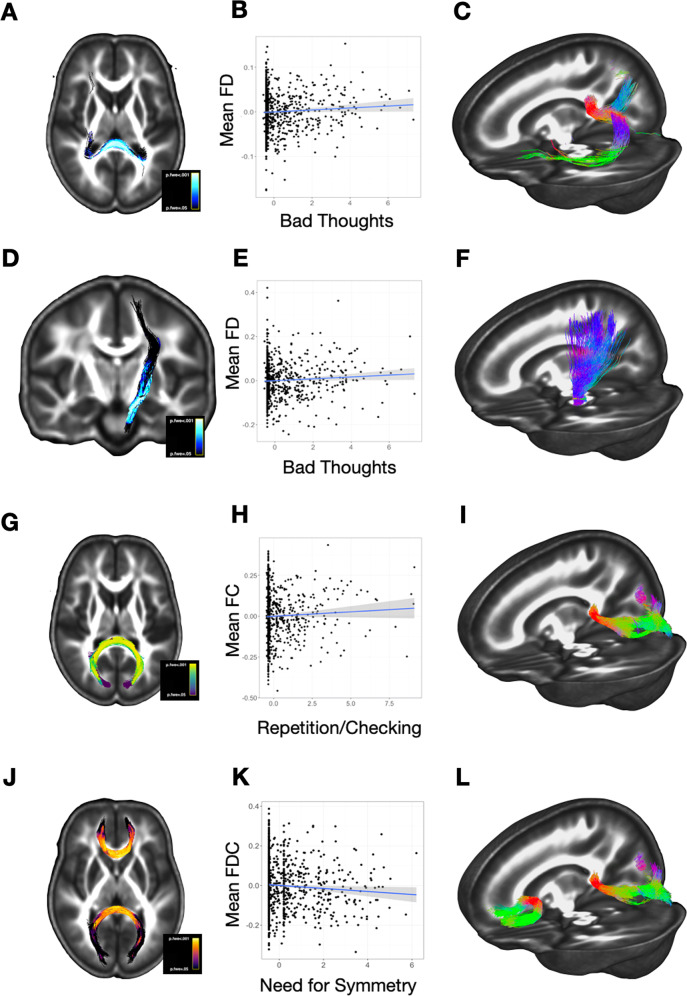
Fixel correlates of OCS dimensions. Panels in the left column (**A**, **D**, **G**, **J**) display the significant regions of association for main OCS-fixel findings. The center column (**B**, **E**, **H**, **K**) presents scatterplots of the assocation between each respective trait (*x*-axes) and the fixel-based value (*y*-axes). Scatterplots of these effects were generated post-hoc, and should not be interpreted as correlations. The right column contains tractograms that further characterize the anatomy of these results (**C**, **F**, **I**, **L**); each significant ROI was used as a seed region and probabilistic tractography was used to construct the tractograms.

We next examined correlates of the four OCS dimensions, in a series of models that contained all four dimensions, the P-factor, age, race, and gender (see Box 3). There was a significant positive association between the Bad Thoughts and FD in the dorsal splenium of the corpus callosum (Fig. 3A–C) and in a portion of the left ascending corticospinal tract that projects through the midbrain (left cerebral peduncle) and internal capsule to sensorimotor regions (*d* = 0.19, p.FWE = 0.04; Fig. 3D–F). There was a positive association between Repetition/Checking and fiber cross-section (FC) in an adjacent region of the splenium (*d* = 0.18, p.FWE = 0.03; Fig. 3G–I). There was a negative association between FDC and Need for Symmetry in two regions of the corpus callosum: in yet another slightly more dorsal region of the splenium, and more anteriorly, in the genu of the corpus callosum (*d* = −0.15, p.FWE = 0.04; Fig. 3J–L). There were no significant associations with the Contamination OCS dimension in brain-wide analysis.

Finally, we ran a series of models incorporating interactions between age and each of the OCS dimensions (3 dependent variables × 4 OCS dimensions = 12 models). None of the OCS × age interactions produced significant effects in these models.

Across all ROI and whole-brain analyses, results did not differ when covarying for history of traumatic stressful life events.

Box 1 The models examining fixel-based (FD, FC, and FDC) correlates of P-factor scores, with all covariates **FD** = P-factor + g factor + age + gender + race + QCmetric

**FC** = P-factor + g factor + age + gender + race + QCmetric

**FDC** = P-factor + g factor + age + gender + race + QCmetric

**FD** = P-factor + g factor + age + gender + race + QCmetric + (P-factor × age)

**FC** = P-factor + g factor + age + gender + race + QCmetric + (P-factor × age)

**FDC** = P-factor + g factor + age + gender + race + QCmetric + (P-factor × age)

Box 2 The models examining fixel-based (FD, FC, and FDC) correlates of OCS factor scores, with all covariates **FD** = P-factor + OCS factor + g factor + age + gender + race + QCmetric

**FC** = P-factor + OCS factor + g factor + age + gender + race + QCmetric

**FDC** = P-factor + OCS factor + g factor + age + gender + race + QCmetric

**FD** = P-factor + OCS factor + g factor + age + gender + race + QCmetric + (OCS factor × age)

**FC** = P-factor + OCS factor + g factor + age + gender + race + QCmetric + (OCS factor × age)

**FDC** = P-factor + OCS factor + g factor + age + gender + race + QCmetric + (OCS factor × age)

Box 3 The models examining fixel-based correlates of the four OCD symptom domain factor scores, with all covariates **FD** = P-factor + Bad Thoughts + Repetition/Checking + Symmetry + Contamination + g factor + age + gender + race + QCmetric

**FC** = P-factor + Bad Thoughts + Repetition/Checking + Symmetry + Contamination + g factor + age + gender + race + QCmetric

**FDC** = P-factor + Bad Thoughts + Repetition/Checking + Symmetry + Contamination + g factor + age + gender + race + QCmetric

### Fractional anisotropy correlates of general psychopathology and OCS

**Fig. 4:**
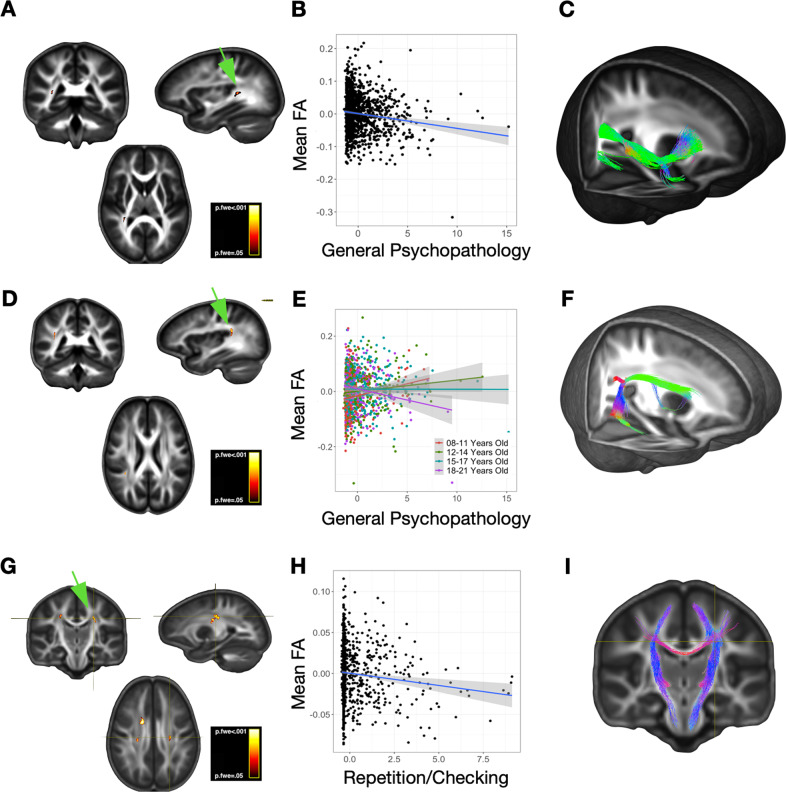
Fractional anisotropy results from whole-brain analyses. The right column displays the localization of significant effects for each trait (**A**, **D**, **G**). The center column presents post-hoc scatterplots with fit lines to illustrate the effects of interest (**B**, **E**, **G**). Note that panel (**E**) shows an age-dependent effect, whereby the lowest quartile age range demonstrated a positive association between p-factor scores and FA, but the highest quartile age range showed an inverse association. Scatterplots of these effects were generated post-hoc, and should not be interpreted as correlations. In the right column, FA results were used to as seed regions to generate probabilistic tractograms (**C**, **F**, **I**), which serve to further characterize the likely pathway(s) intersecting the localization of the FA findings.

FA findings were spatially distinct from the results of our fixel-based analyses. FA was negatively associated with general psychopathology (p-factor), with a main effect in the right inferior longitudinal fasciculus (*d* = −0.13, p.FWE = 0.03; Fig. 4A) and a p-factor × age effect in the right superior longitudinal fasciculus (*d* = 0.01, p.FWE = 0.02; Fig. 4B). Streamlines originating from these regions followed trajectories that were quite distinct from those identified in fixel-based analyses (compare especially Fig. 2C, 4F). Interestingly, the nature of the p-factor × age interaction on FA was similar to the corresponding interaction in our whole-brain fixel analysis, even though the brain region was distinct: there was a positive correlation between p-factor and FA in the youngest quantile, but a negative correlation in the oldest (compare Figs. 2B, 4D).

FA was also negatively associated with Repetition/Checking in bilateral clusters of white matter in the centrum semiovale, including portions of the corpsus callosum and CST (*d* = −0.21, p.FWE = 0.01; Fig. 4G–I). Streamlines from the corpus callosum cluster fed into both the corpus callosum and the corticospinal tracts, a pattern distinct from that seen in fixel-based analysis (compare Figs. 2I, 4I). There were no FA associations with the other OCS dimensions (Bad Thoughts, Symmetry, Contamination), and there were no significant interactive FA effects between any of the OCS dimensions and age.

### Nonlinear effects of age on p-factor and OCS

Exploratory whole-brain analyses incorporating nonlinear age effects are included as supplementary materials (Supplementary Table 5 and Supplementary Fig. 3). Results were unchanged from the original models, with two exceptions. First, there was a newly significant interaction between age^2^ and Repetition/Checking on FC in the left external capsule (*d* = −0.02, p.FWE = 0.03); simple effects plots revealed that the youngest and oldest age groups showed an inverse association between FC and Repetition/Checking, but this association was not evident in the middle age range of the present sample (Supplementary Fig. 6). Second, the age-by-p-factor contrast (Fig. 2) was no longer significant when a quadratic term for age was used.

### Results for age and cognition

We performed exploratory whole-brain fixel-based analyses of the white matter correlates of age (linear and quadratic) and of cognition in this sample. These are presented in the supplementary materials (Supplementary Tables 6, 7; Supplementary Figs. S4, 5). Relationships between cognitive measures and OCS were minimal, and not statistically significant; these are shown in Supplementary Fig. S6.

## Discussion

We examined the white matter correlates of OCS and of general psychopathology in a large, representative sample of children, adolescents, and young adults, using both FA and fixel-based analyses. We found no significant associations between white-matter measures and OCS in any of the ROIs identified a priori based on previous reports using FA. However, exploratory whole-brain analyses, robustly corrected for multiple comparisons, identified white matter disruptions associated with several OCS dimensions and with the interaction between general psychopathology and age. These abnormalities in structural connectivity may relate to both general/transdiagnostic and OC symptom-specific neural network dysfunction.

We first examined the white matter correlates of general psychopathology. P-factor scores were associated with fiber density in the splenium of the corpus callosum in an age-dependent manner: psychopathology was associated with higher fiber density in younger adolescents/children, but lower fiber density appeared in the oldest age group. The effect was localized in a medial region of the splenium that connects the bilateral occipital lobes via the forceps major. This age-dependent association may reflect early hypermaturation followed by a later growth deficit [41], a pattern that was also identified in cortical morphology data from this sample [30]. The section of the splenium where the effect was strongest undergoes accelerated maturation around the time of puberty [42], lending support to the notion that developmental dysregulation during this sensitive window may contribute to network dysfunction and subsequent psychopathology risk.

It is striking that the general psychopathology factor (Fig. 2) and the OCS dimensions of Bad Thoughts, Repetition/Checking, and Symmetry (Fig. 3A, G, J) are all associated with white-matter abnormalities in the posterior corpus callosum. Closer examination revealed that the affected regions are not identical (Supplementary Fig. 3). The correlate of the Bad Thoughts dimension is more dorsal in the corpus callosum than the other factors, with corresponding seed-generated white matter streamlines extending into the dorsal parietal lobe. In contrast, the white-matter regions associated with the Repetition/Checking and Symmetry dimensions are more ventral, with streamlines extending into the posterior parietal/occipital lobe, and are difficult to distinguish from the region associated with the interaction of general psychopathology with age. The Symmetry OCS dimension is also associated with anterior corpus callosum abnormalities and is the only psychopathology dimension to demonstrate associations with any frontal white matter region.

It is notable that we did not observe any significant associations between general psychopathology and fiber metrics (including FA) in frontal or frontotemporal pathways. This contrasts with a previous report of an analysis conducted in a subsample of this same dataset [9]. This inconsistency may be due to substantial method variance, since Alnæs et al. [9] examined ICA-derived white matter “factors” based on the covariation of multiple microstructural properties in multiple pathways. In contrast, we attempted to delineate specific fiber properties in specific pathways (FD, FC, FDC), and would therefore not have captured the composite frontotemporal “crossing/complexity” variation that Alnæs et al. [9] report as linked to psychopathology scores.

Our findings dovetail with a recent report of an inverse association between genetic (SNP-based) psychopathology risk and forceps major white matter connectivity in a developmental sample (the PING study; *N* = 678) and in adults from the UK Biobank sample [43]). This suggests that developmental alterations in forceps major structural connectivity are at least partially attributible to additive inherited risk for psychopathology, across samples and across diagnoses.

After controlling for the effects of general psychopathology, we identified several associations between OCS and white matter metrics. White-matter abnormalities in the splenium, overlapping with those identified for general psychopathology, were also associated with the OCS dimensions of Repetition/Checking and Need for Symmetry. Interestingly, the Repetition/Checking was positively associated with fiber cross-section measure (FC), while Need for Symmetry was negatively associated with the combined cross-section/density metric, FDC. This suggests that these three effects—those of p-factor × age, Repetition/Checking, and Need for Symmetry—are related to subtly different abnormalities in white matter, even though the effects spatially overlap.

Finally, the Bad Thoughts dimension was correlated with fiber density (FD) in a more dorsal region of the splenium that connects the bilateral precuneus and bilateral temporal lobes, raising the possibility that alterations to these white matter pathways disrupt networks that coordinate self-related or self-reflective processing. Severity of Bad Thoughts was also negatively associated with FD in a portion of the left corticospinal tract that extends from the left cerebral peduncle through the internal capsule and terminates dorsally in sensorimotor cortices. Alterations in this pathway could disrupt motor and/or somatosensory processing, highlighting the potential role of somatosensory inputs (including perhaps proprioceptive or visceral inputs) in triggering aversive thoughts.

The convergence of several results in the splenium of the corpus callosum raises the possibility that slight deviations in the rapid growth and myelination of the splenium during adolescence [42] may result in various patterns of network dysfunction, ultimately affecting downstream variation in vulnerability for OCD-relevant symptom profiles. Recent large-scale data-driven studies of functional [44] and structural [43, 45, 46] imaging data increasingly implicate visual association cortices and somatosensory-motor connectivity in general psychopathology risk. Repetition/Checking and Need for Symmetry are associated with abnormalities in occipital interhemispheric pathways; we speculate that structural deficits in those pathways may alter hemispheric synchronization of lower-level visual processing and integration. These alterations in visual information processing could, in turn, create a predisposition for unstable internal representations [47] or heightened error monitoring related to sensory information [48], perhaps contributing to a heightened sensitivity to visual symmetry and/or dysregulation of perceptual memory (the latter of which may drive some types of checking/repeating) [49].

We found no significant associations with a general OCS factor score that captured shared variance across all 16 OCS items. This suggests that the structural correlates of distinct dimensions of OCS are meaningfully different. Consistent with this idea, a recent hierarchical taxonomy of psychopathology (HiTOP) report suggested that some OC symptoms are most correlated with a “fear” spectrum while others had substantial cross-loadings on a “thought disorder” spectrum [50].

When we analyzed FA correlates of OCS, three areas of significant effect were identified, but the results were distinct from those found in fixel-based analyses (Fig. 4). Interestingly, in two of these areas, fixel-based representations of the local anatomy showed prominent crossing fiber architecture. This pattern suggests that FA values in these clusters may be influenced by local anatomical properties other than the microarchitecture of any specific fiber bundle that traverses the identified regions. Possibilities include the microarchitectural interaction of crossing pathways, inflammation/free water, or other extracellular/microcellular features within the identified clusters. The lack of alignment between FA and our newer fixel-based analysis confirms that these two approaches are sensitive to different aspects of white-matter organization. The relationship between FA and fixel-based measures in selected regions is shown in Supplementary Fig. S7.

We did not identify any white matter abnormalities in our primary analysis of 14 ROIs derived from previous literature. There are two possible reasons for this. All previous studies of white matter in OCD/OCS have used FA, rather than fixel-based analysis. Since FA and fixel-based analyses identify different aspects of white matter dysregulation, ROIs based on past FA studies may not be optimal for the identification of fixel-derived white matter abnormalities. Alternatively, the regions of white matter abnormality associated with OCS may be more focal than the anatomical ROIs identified in past literature. A large ROI may include foci of symptom-associated white-matter disruption within a substantial volume of normal white matter; meaningful OCS-related differences may be diluted in aggregate measure from full ROIs. This suggests that voxel-wise, cluster-corrected exploratory analyses may be more sensitive, which is precisely what we observe here.

Although several of our results were localized in regions that overlap with recent evidence from large multimodal imaging studies of OCS/OCD in adolescence [13, 23], it is worth noting that our findings are not aligned with predominant contemporary models that link OCD pathology to dysconnectivity in fronto-striatal pathways [15, 51]. Given the present evidence that at least some domains of psychopathology are linked to white-matter alterations in an age-dependent manner, it is possible that developmental risk signatures of OCS are distinct from those of OCD pathophysiology in adulthood.

### Limitations and future directions

Strengths of the present study include the sample size, the fixel-based diffusion analytic technique, and the use of factor-derived scores to model both general psychopathology and dimensions of OCS. By focusing on dimensional symptom scores in a young, mostly subclinical sample, we avoid confounds related to chronicity and medication. However, there are several important limitations. First, a detailed analysis of race/ethnicity and social determinants of health was beyond the scope of this report. While we did include race as a covariate in all analyses, there is evidence that self-reported race captures complex aspects of social and historical dynamics that contribute to variation in chronic stress and environmental exposures, and that this variation in lived experience has important consequences for neural development in the socioemotional and cognitive domains [52]. Teasing apart the influence of early life experiences and exposures linked to race will be an important goal of future research. Another limitation is that the DWI acquisition was not optimized for fixel-based analysis. The constrained spherical deconvolution algorithm that forms the basis of the fixel-based pipeline functions optimally at higher b-values (*b* = 2000 or greater) and/or with multiple diffusion shells, and it is possible that the use of standard clinical MRI b-values (*b* = 1000) may have attenuated the signal-to-noise ratio in the present study. However, there is evidence that even at *b* = 1000, the fixel-based approach outperforms tensor-based approaches in ground truth analyses of fiber properties [28]. While the current approach is developmentally informative, the cross-sectional design limits interpretation in a developmental context, and future studies should examine the interplay of psychopathology and white matter signatures in a longitudinal framework. We assumed a higher-order p-factor in our modeling approach and examined additive variance in the white matter correlates of higher- and lower-order factors. Other (nonadditive) relationships are possible, and even larger samples would be needed to perform exploratory analyses that arbitrate among the myriad possible nonadditive relationships. Finally, while the effects we report are statistically robust, surviving correction for multiple comparisons, the effect sizes are small; they may be informative to generate new insights into developmental pathophysiology, but they are far from what would be needed to be clinically actionable.

In conclusion, we show that white matter disruptions linked to general psychopathology are evident in early adolescence and are age-dependent. In addition, we describe an OCS dimension-specific pattern of white matter alterations in pathways supporting interhemispheric processing as well as visual and sensorimotor integration. These findings suggest that distinct dimensions of OCD-spectrum psychopathology can be delineated from an index of transdiagnostic psychopathology risk and are dissociable at the level of white matter biomarkers. This work has implications for explaining how specific neural network disruptions in adolescence contribute to heterogeneity in OCD-spectrum psychopathology.

## Supplementary information

Supplemental Methods and Tables

Figure S1

Figure S2

Figure S3

Figure S4a

Figure S4b

Figure S5a

Figure S5b

Figure S6

Figure S7

Supplemental Materials – Table of Contents

## Data Availability

All code and scripts used to perform these analyses will be made available upon request to the corresponding author.
