# Supplementary material for: Obsessive compulsive symptom dimensions are linked to altered white-matter microstructure in a community sample of youth
Source: Transl Psychiatry. 2022 Aug 10;12:328. doi: 10.1038/s41398-022-02013-w (PMC9365814; doi:10.1038/s41398-022-02013-w)

*DWI acquisition and preprocessing using MRtrix3*

Following dcm2nii conversion, data were preprocessed according to the MRtrix3 fixel-based workflow. Images were visually inspected by an expert (RGG) and excluded on the basis of venetian blind artifacts, gross signal hyperintensities, and excessive slice dropout. Based on this visual inspection, 89 datasets were excluded. The remaining datasets were denoised (Veraart et al., 2016), eddy-induced distortion-corrected (via FSL’s EDDY), and then bias field corrected. Based on the QC data generated by EDDY, additional participants were excluded on the basis of mean framewise displacement (mean displacement > 1.5mm for both DWI acquisitions). 14 participants were excluded on the basis of excessive head motion and 1 participant was excluded on the basis of contrast-to-noise ratio (CNR) less than 0.25). Generally speaking, CNR was good-to-excellent. We referred to the diffusion QC guidelines established by Roalf et al. (2016) in this same sample.

Corrected images were upsampled to a voxel size of 1.3mm isometric. Within Mrtrix3, b=0 images can be used as a second “shell” from which CSF-specific response functions are estimated in each participant. This calculation allows for “multi-shell, multi-tissue” deconvolution of the diffusion signal using single-shell data, which enhances the signal from white matter relative to free water (Dhollander, Raffelt, & Connelly, 2016). Per-subject tissue-specific response functions were computed, and then averaged across subjects to create sample-specific response functions for white matter and CSF, respectively (Dhollander et al., 2016).

Constrained spherical deconvolution was performed for white matter, and the resulting white matter fiber orientation distribution (FOD) images were then co-registered to create a sample-specific FOD analysis template. To reduce computation time, the FOD template was constructed using a subsample of 40 individuals that was representative of the full sample in terms of age, race, and sex. Peak FOD amplitudes at each fixel in this template were calculated and thresholded (at .30) to remove fixels containing residual gray matter peaks. This thresholded image was then used to create an “analysis fixel mask” which represents the co-registered fixel-based white matter template across all subjects in the sample. Individual subjects’ FOD images were then transformed into template space. Fixel-based analysis generates three measures of white matter integrity: fiber density (FD), fiber bundle cross-section (FC), and combined fiber density and cross section (FDC; see Supplemental Figure S1). FD captures the volume of the intra-axonal restricted compartment, while FC is a more macrostructural approximation of the relative white matter bundle size (relative to the study population); FDC is simply, a combined measure that modulates density by cross-section (Raffelt et al., 2016). Final per-subject fixel images for FD were computed by taking each fixel in reoriented subject space and assigning it to the corresponding fixel in template space. Final FC images were computed by extracting the subject-to-template difference at each fixel and log transforming these difference scores for use in analysis. FDC was computed for each subject by multiplying FD fixel values by FC fixel values. A whole brain tractogram was generated using fod template space (tckgen), and tcksift was subsequently used to reduce biases in the tractogram by using the underlying spherical harmonics to retain the top 200,000 most robust streamlines. This final tractogram was used to inform fixelcfestats anatomical clustering in all whole brain fixel analyses.

Subject-wise diffusion tensors and subsequent FA maps were computed using the preprocessed and upsampled DWI images. These FA images were warped to the FOD-based analysis template space employed in the fixel-based analysis in order to maximize anatomical overlap with the fixel-based findings.

*ROI-based analyses*

ROIs were generated by an expert analyst (RGG) using probabilistic tractography in the MRtrix3 framework. Given that the final list of ROIs were derived from several different a priori sources, a mix of approaches was taken to maximize anatomical overlap between ROIs reported in earlier studies and the final ROIs used in the present analyses. For the cingulum bundles and corpus callosum, tractography was based on published tractography/histological atlases (Aboitiz, Scheibel, Fisher, & Zaidel, 1992; Haber et al., 2020; Wakana, Jiang, Nagae-Poetscher, van Zijl, & Mori, 2004); for these pathways, seed-and-target regions were placed on the common fod template according to Wakana et al. (2004), and subsequent tractograms were verified against the published atlases. For the uncinate fasciculi, the thalamic radiations, and the saggital stratum, tractography was based on the JHU ICBM white matter atlas (per the findings of Piras et al., 2021), and using the JHU atlas and registering/warping each voxel-based JHU ROI to template space. Each of these voxel-based JHU ROIs was converted to fixel space for the fixel ROI analyses. For the left superior corticostriatal tract, tractography was based on the tractography manual included with the AtlasTrack software employed by Pagliaccio et al. (2021; (Hagler et al., 2019). The OFC pathway we included is not a part of major white matter histological atlases; for this novel OFC pathway, seeds were placed in the L and R anterior limb of the internal capsule (respectively) with target regions encompassing the ventral half of a single coronal slice of the frontal lobe. Only one coherent pathway emerged (per hemisphere) in this exploratory OFC pathway generation step. All tractography-generated ROI pathways were converted to the common template fixel space using the tck2fixel command, and subsequently converted to voxel space using the fixel2voxel command.

Generating tractograms using a non-standardized template is not an exact science and involves an iterative process of trimming and editing initial (literature/anatomy-informed) seed, target, and waypoint ROIs such that the final tractogram matches the published anatomical literature and/or meets individualized project goals. The majority ROIs were established and refined using atlases and/or existing published manuals that inform ROI placement and final anatomy. We have provided figures that show the anatomy and location of all the ROIs that were generated, in order to allow readers to visually verify tract placement. The exact code for the construction of the novel OFC pathway is shown below, along with an axial slice figure demonstrating seed and target placement. Full code for all ROI pathways available upon request to the corresponding author.

## construct initial OFC pathway with a minimum fd cutoff of 0.2, a maximum length of 40, and

## a maximum streamline selection of 500

tckgen -force -select 500 -maxlen 40 -cutoff 0.2 -seed_image OFC_WM_02_L.mif ../wmfod_template.mif OFC_WM_03_L_cutoff2_maxlen40_500.tck

tckgen -force -select 500 -maxlen 40 -cutoff 0.2 -seed_image OFC_WM_02_R.mif ../wmfod_template.mif OFC_WM_03_R_cutoff2_maxlen40_500.tck

## after verifying the pathways visually in mrview, convert tractography files to

## binary fixel masks in the population-specific fixel space

tck2fixel OFC_WM_03_L_cutoff2_maxlen40_500.tck ../fixel_mask OFC_WM_03_L_cutoff2_maxlen40_500/OFC_WM_03_L_cutoff2_maxlen40_500.mif

tck2fixel OFC_WM_03_R_cutoff2_maxlen40_500.tck ../fixel_mask OFC_WM_03_R_cutoff2_maxlen40_500/OFC_WM_03_R_cutoff2_maxlen40_500.mif

## convert fixel masks to voxel masks for FA analyses using the "max" option

fixel2voxel OFC_WM_03_L_cutoff2_maxlen40_500/OFC_WM_03_L_cutoff2_maxlen40_500.mif max OFC_WM_03_L_cutoff2_maxlen40_500/OFC_WM_03_L_cutoff2_maxlen40_500_mask_voxel.mif

fixel2voxel OFC_WM_03_R_cutoff2_maxlen40_500/OFC_WM_03_L_cutoff2_maxlen40_500.mif max OFC_WM_03_R_cutoff2_maxlen40_500/OFC_WM_03_L_cutoff2_maxlen40_500_mask_voxel.mif


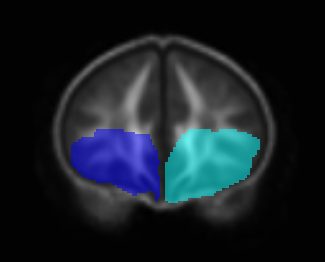

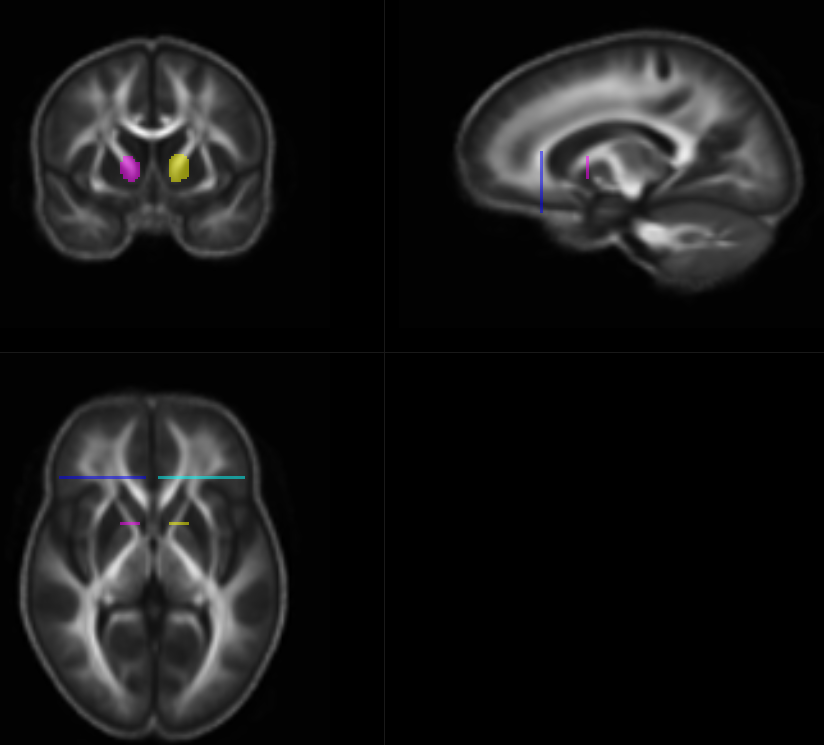

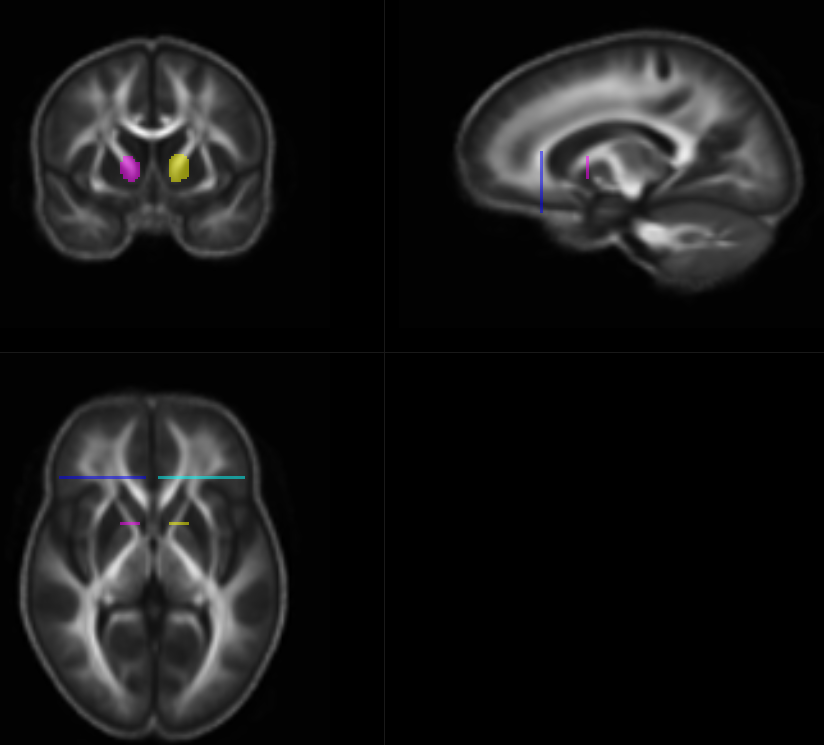


*Comparison of included vs. excluded participants*

Of the participants with complete MRI data and complete OCS item data, 1,208 were included for the final analyses and 97 were excluded because their diffusion data did not pass QC screening (see details in the Supplemental Methods, above). Excluded participants did not differ from the included sample on sex ratio (*X^2^(1)* = .44,p-value = .507), proportion of reported Black (*X^2^*(1)= 3.2557, p = .07118 ) or Other-reported race (*X^2^*(1) = 0.88582, p = .3466), p-factor scores (t(106.65) =.27, p = .79), OCS factor scores(t = -.01 to -.29, p = .43 to .98) or maternal years of education (t(110.33 = -1.1356, p = .2586). Included participants were significantly younger than the excluded participants (t(110.12) = -3.1901, p = .002) and had significantly higher g-factor scores (t(109.24) = -4.2529, p-value = .00004).

**Details of model choice and assumptions**

The OCS dimensions were derived in a way that maximizes their independence, but they are still moderately correlated (0.52-0.57). All correlations and histograms are presented in Figure 1. These correlations do not necessarily imply problematic collinearity. To check the influence of collinearity in models that included all symptom scores as covariates, we calculated the variance inflation factor for each predictor. VIF for the four OCS dimensions and the p-factor dimension ranged from 2.0-3.1, suggesting a mild-to-moderate level of variance inflation. As a rule of thumb, VIF is of potential concern when values fall between 5 and 10, and of serious concern when VIF is 10 or greater. Therefore, we have reason to believe that multicollinearity is not a serious issue in our analyses.

Visual inspection of our ROI model residuals (Q-Q plots and residuals vs. leverage) suggested normality of the residuals (see images below for a few examples of these plots).

Model fit can also be problematic when the DV is skewed, however, our DVs *are* normally distributed in our ROI data (illustrated in Figure S6). For the whole brain fixel and voxel analyses, the nonparametric nature of the whole brain GLM analyses is equally robust to both normally and nonnormally distributed data.

***Sample plots to support normality of residuals:***

**Cingulum FD (Left) = symptoms +covariates**


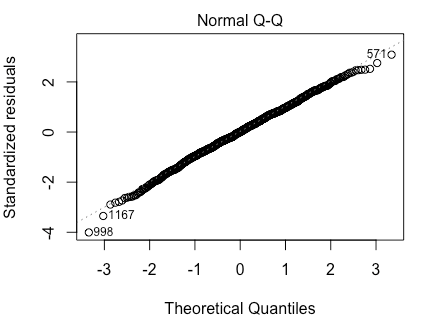

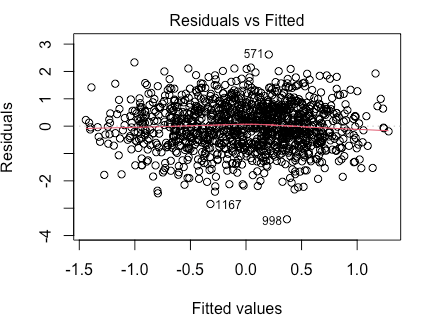


**CC Body FD = symptoms + covariates**


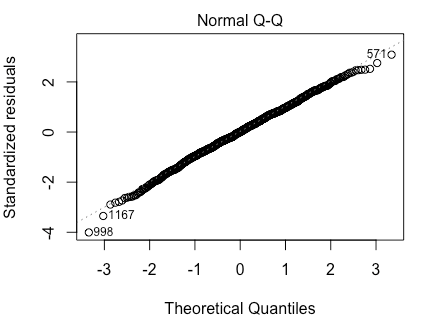

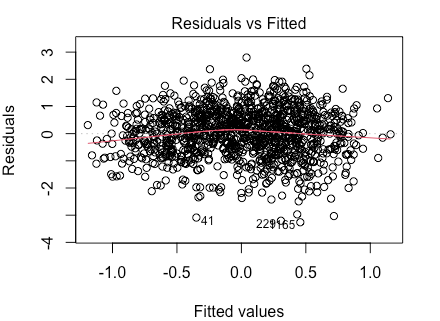


**Orbitofrontal path FC (Right)= symptoms +covariates**


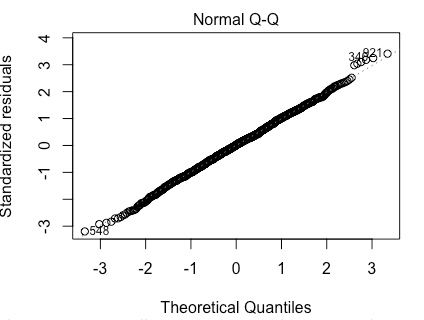

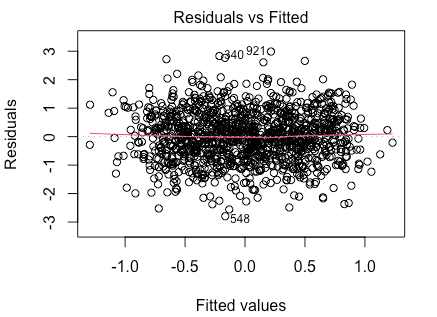

Supplement: Supplementary file 1 — Supplemental Methods and Tables [file 41398_2022_2013_MOESM1_ESM.docx]
