# Supplementary figures and images for: Obsessive compulsive symptom dimensions are linked to altered white-matter microstructure in a community sample of youth
Source: Transl Psychiatry. 2022 Aug 10;12:328. doi: 10.1038/s41398-022-02013-w (PMC9365814; doi:10.1038/s41398-022-02013-w)

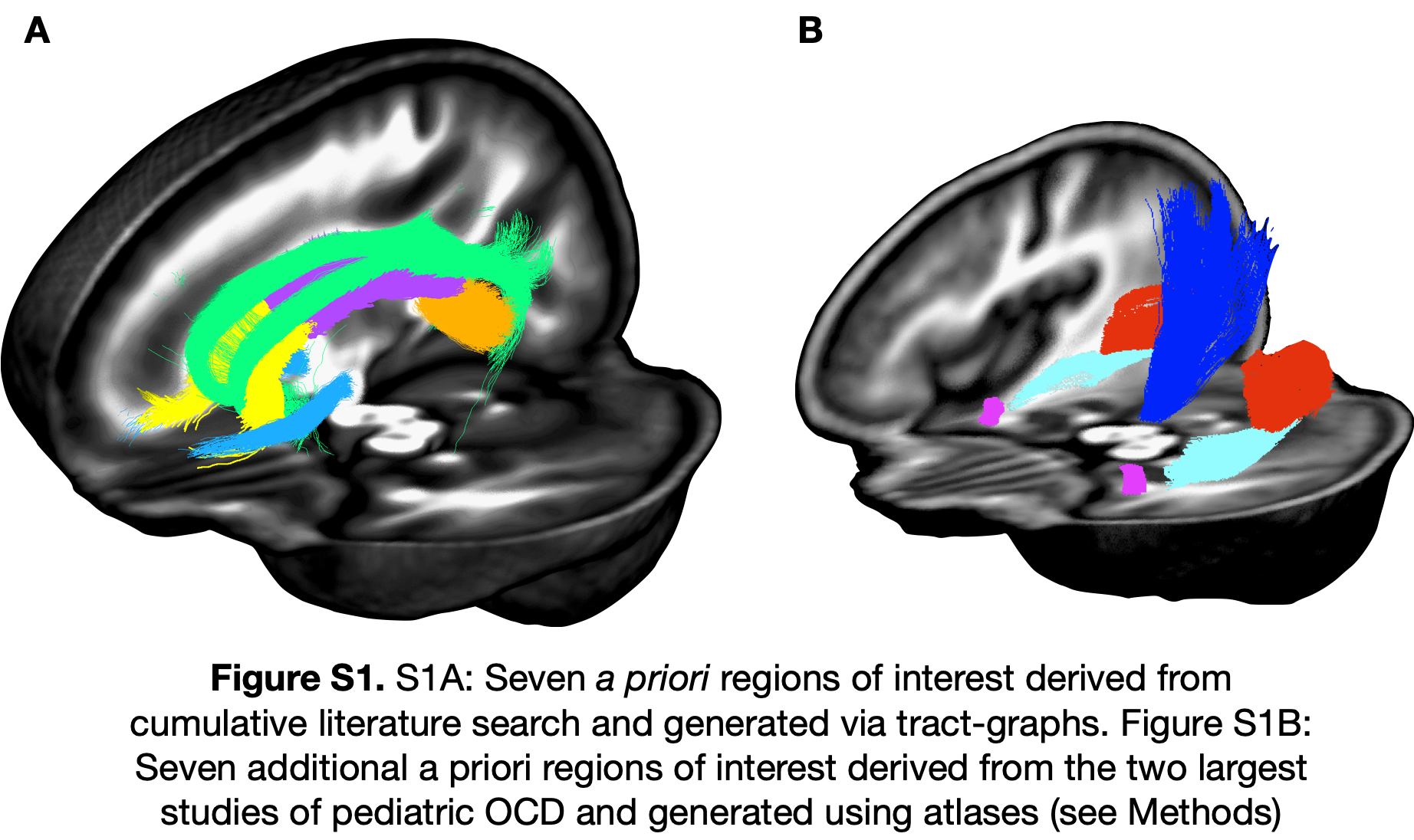

Supplement: Supplementary file 2 — Figure S1 [file 41398_2022_2013_MOESM2_ESM.jpg]

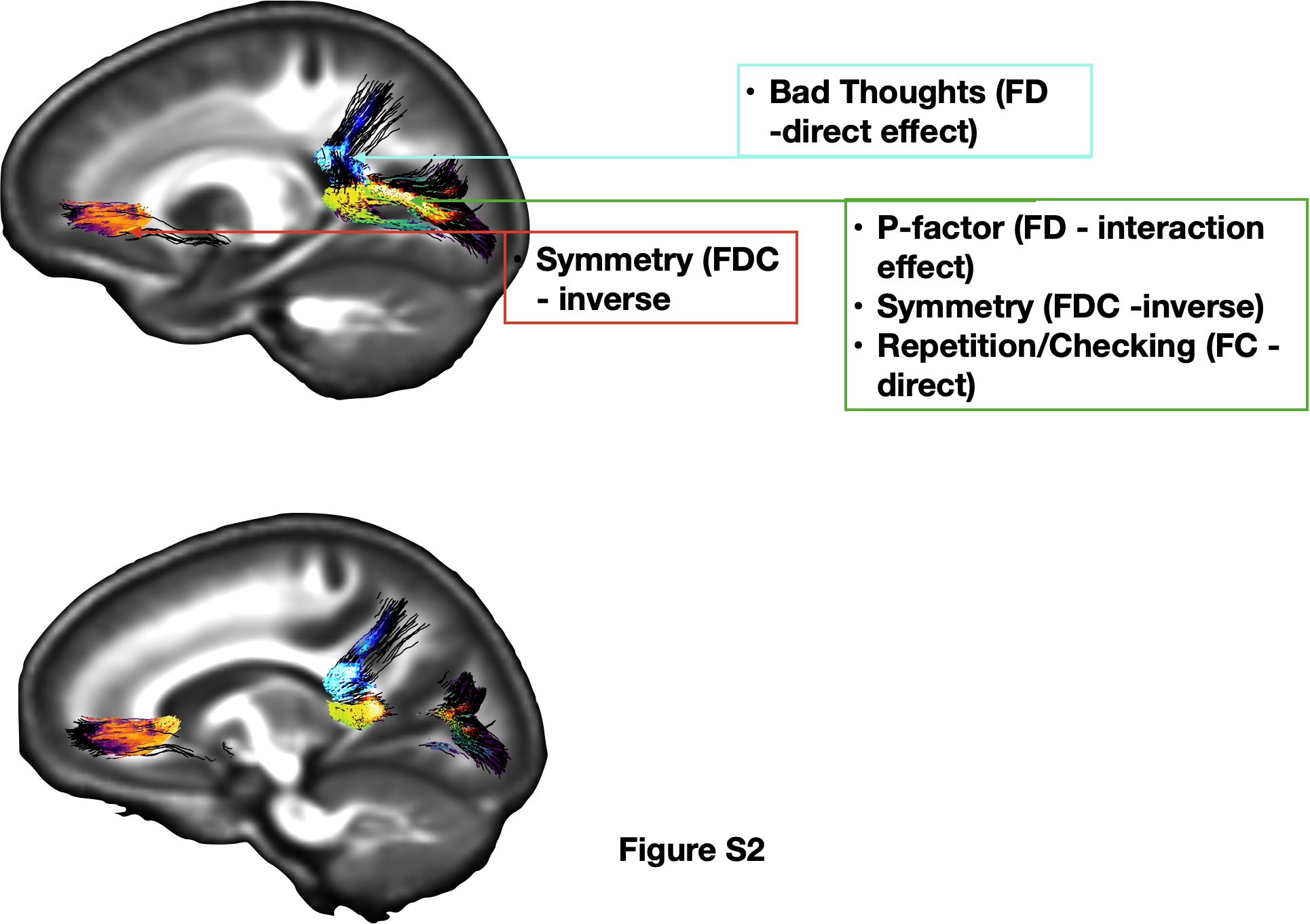

Supplement: Supplementary file 3 — Figure S2 [file 41398_2022_2013_MOESM3_ESM.jpg]

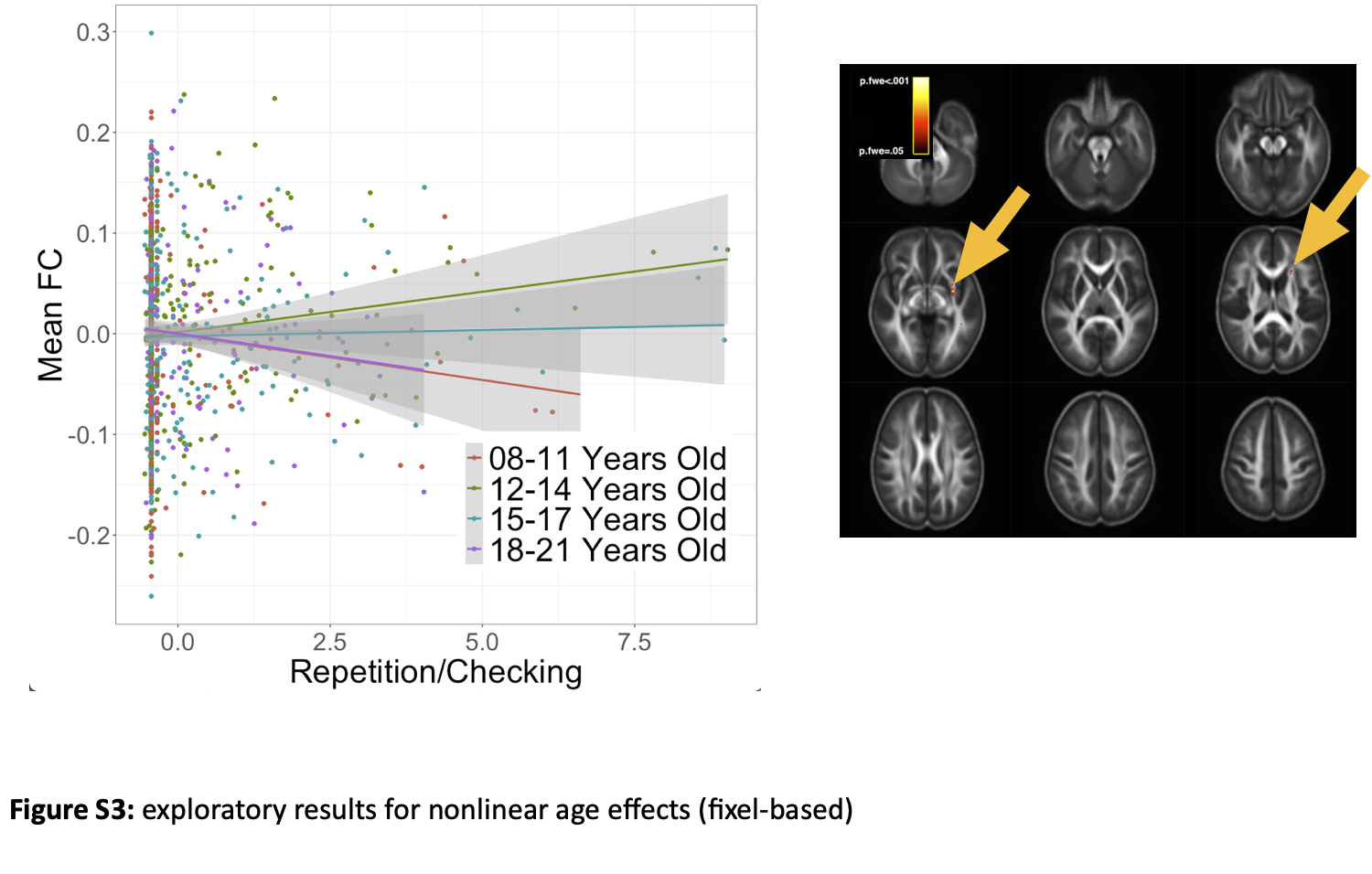

Supplement: Supplementary file 4 — Figure S3 [file 41398_2022_2013_MOESM4_ESM.jpg]

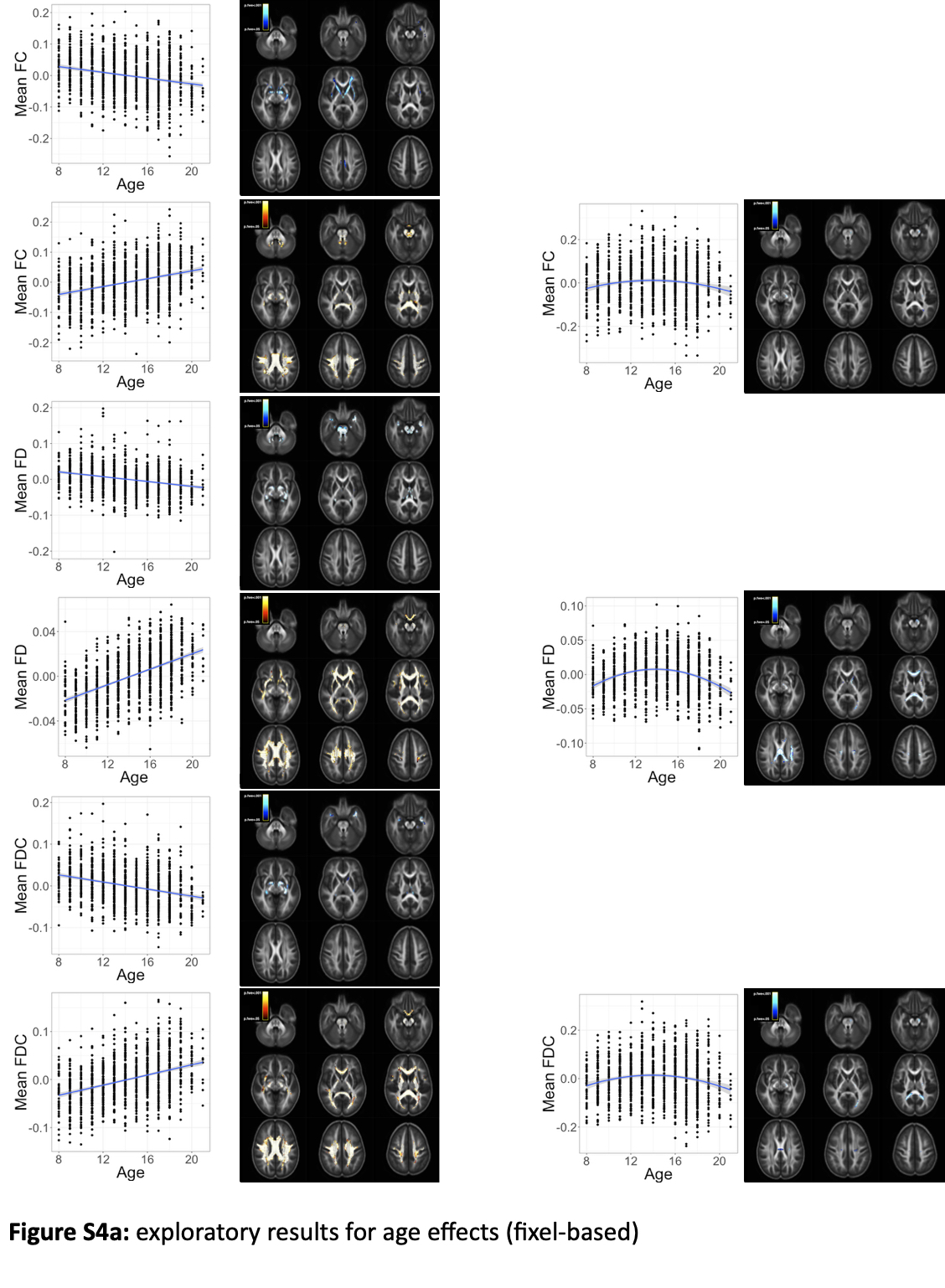

Supplement: Supplementary file 5 — Figure S4a [file 41398_2022_2013_MOESM5_ESM.jpg]

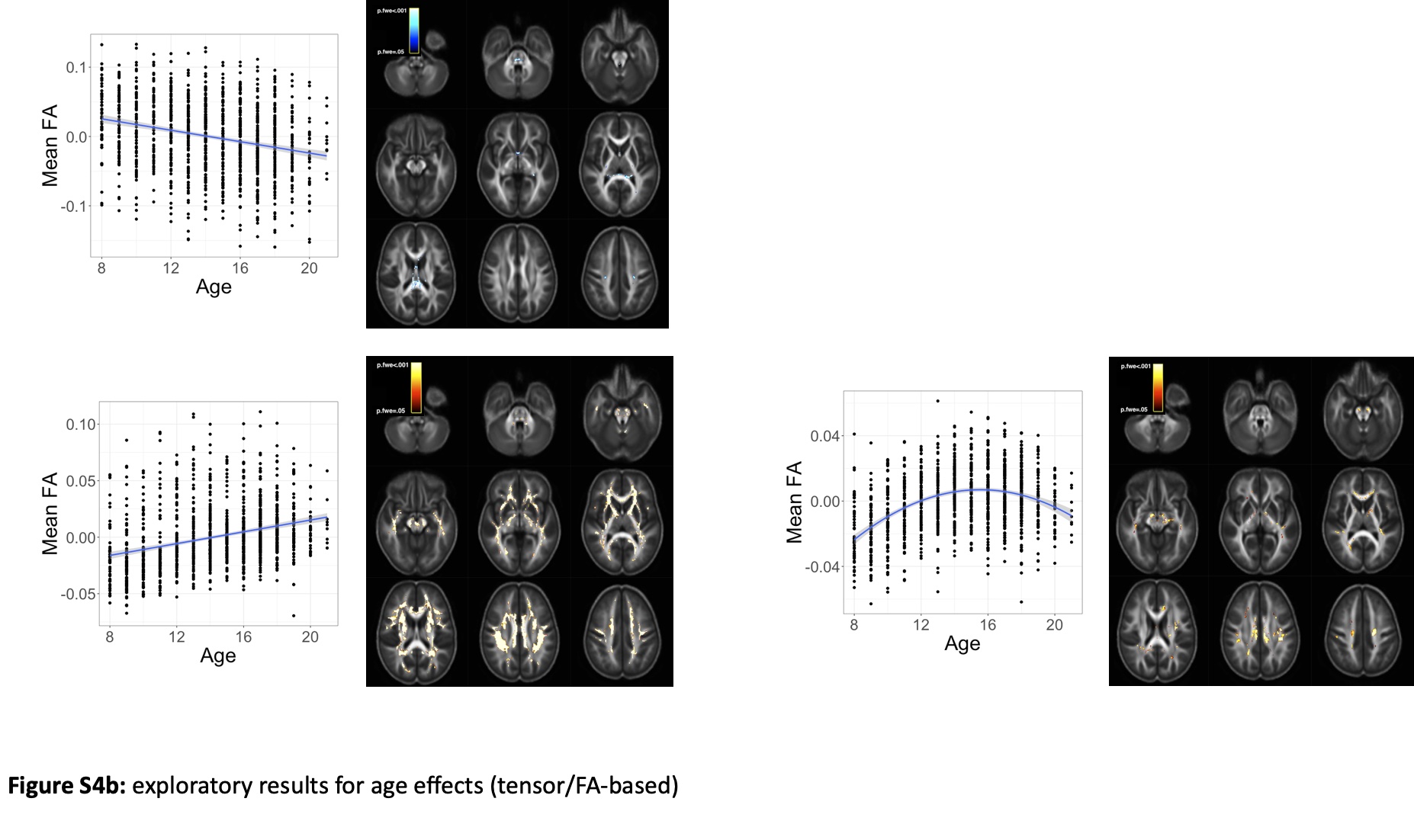

Supplement: Supplementary file 6 — Figure S4b [file 41398_2022_2013_MOESM6_ESM.jpg]

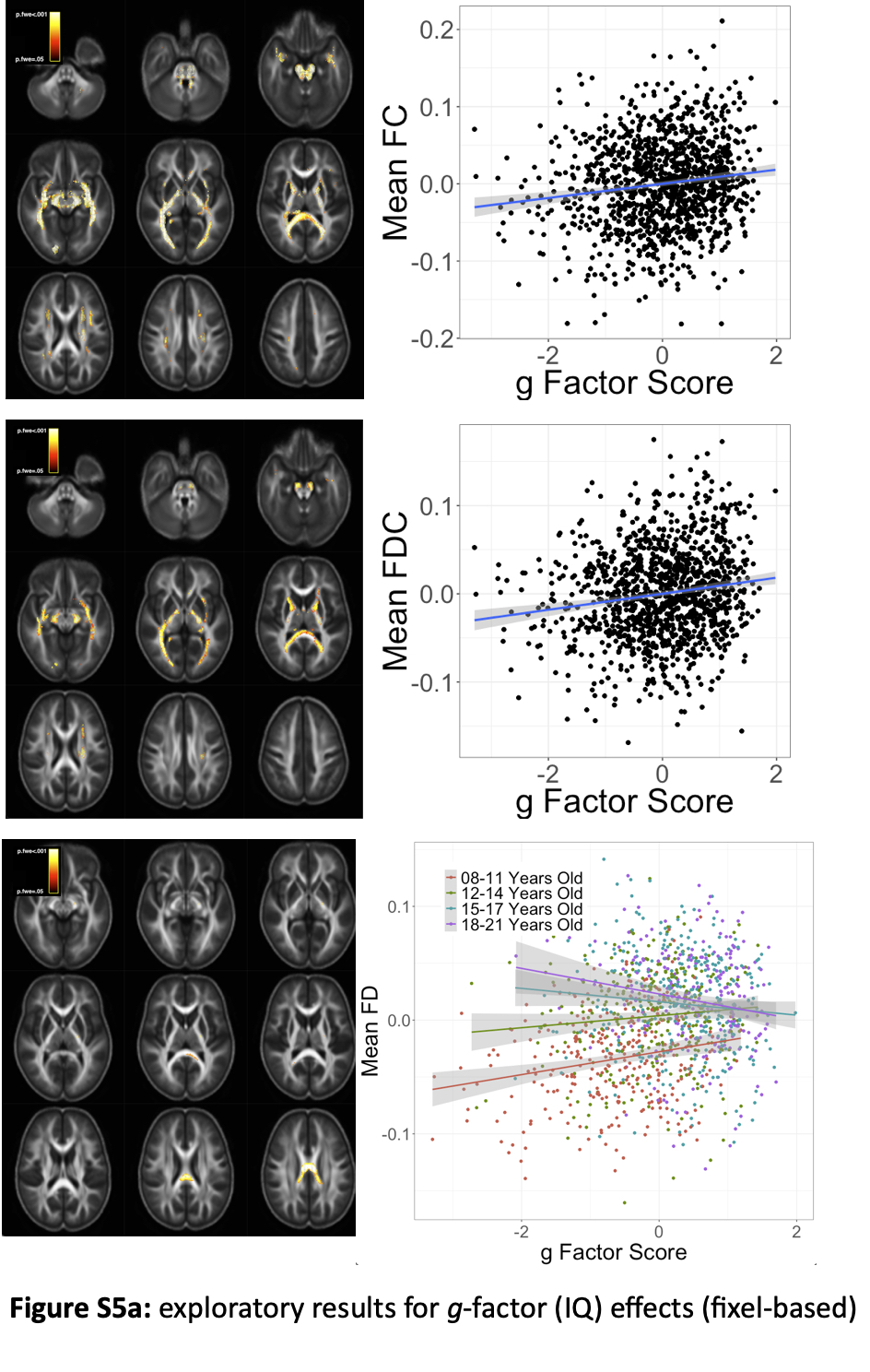

Supplement: Supplementary file 7 — Figure S5a [file 41398_2022_2013_MOESM7_ESM.jpg]

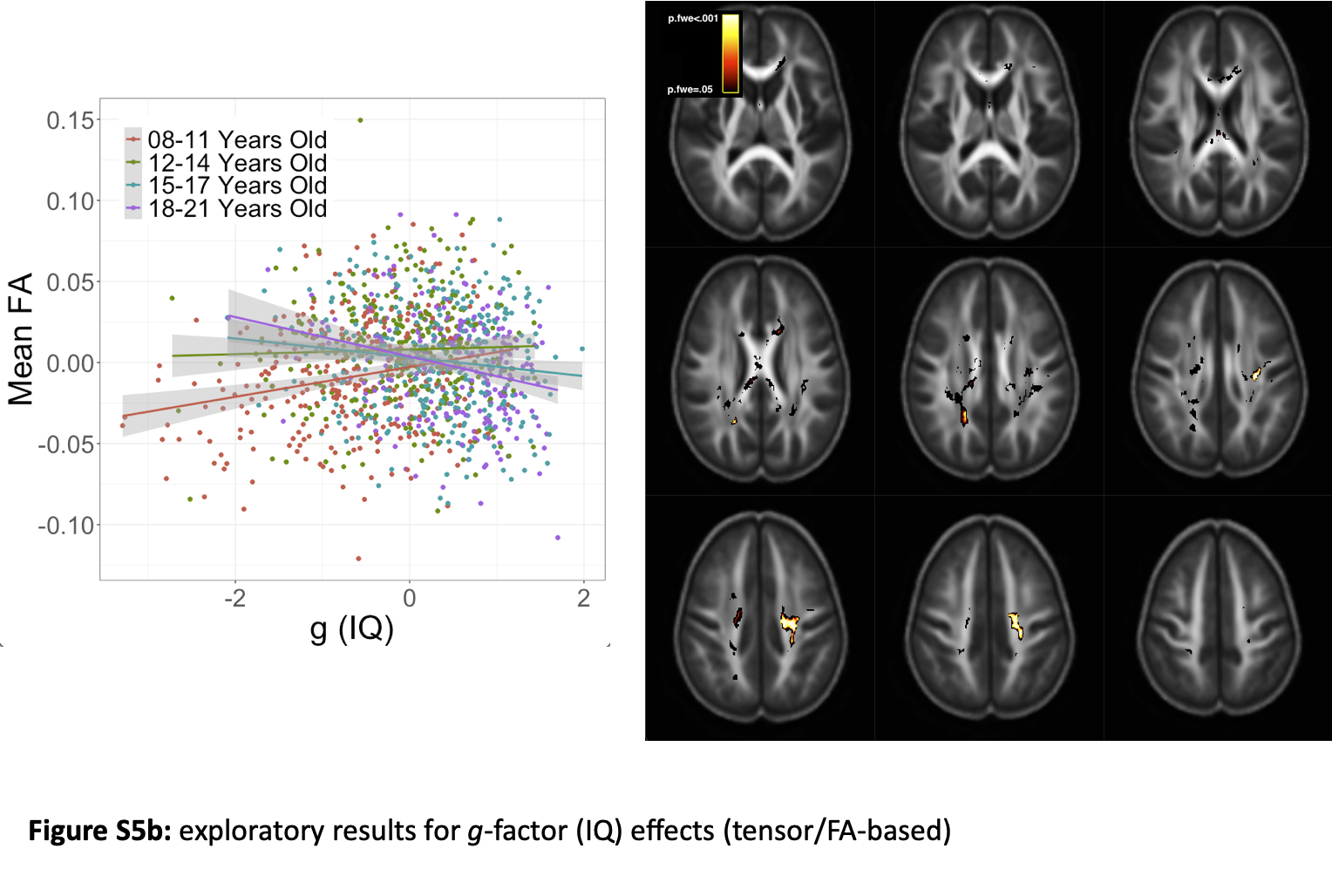

Supplement: Supplementary file 8 — Figure S5b [file 41398_2022_2013_MOESM8_ESM.jpg]

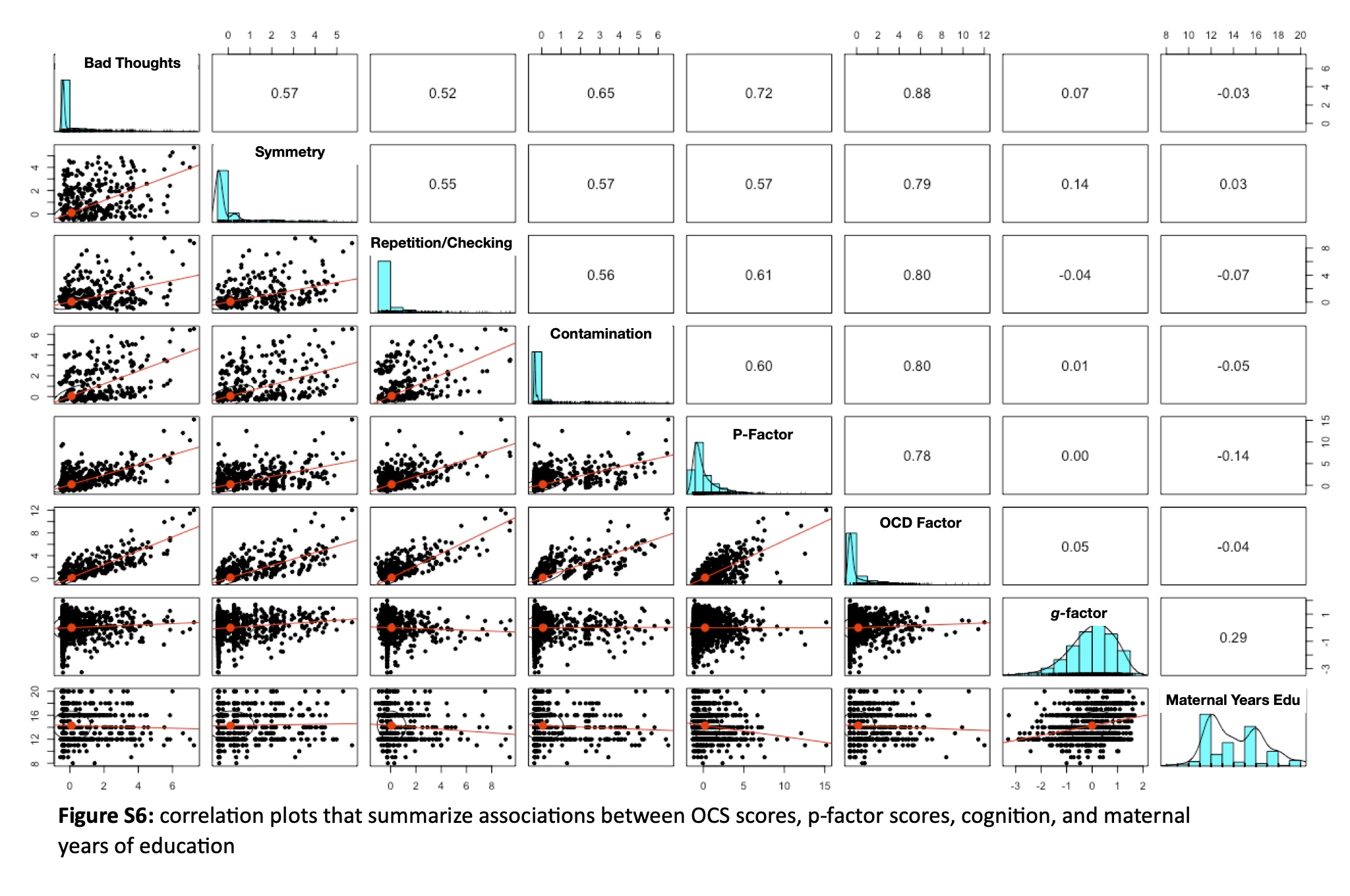

Supplement: Supplementary file 9 — Figure S6 [file 41398_2022_2013_MOESM9_ESM.jpg]

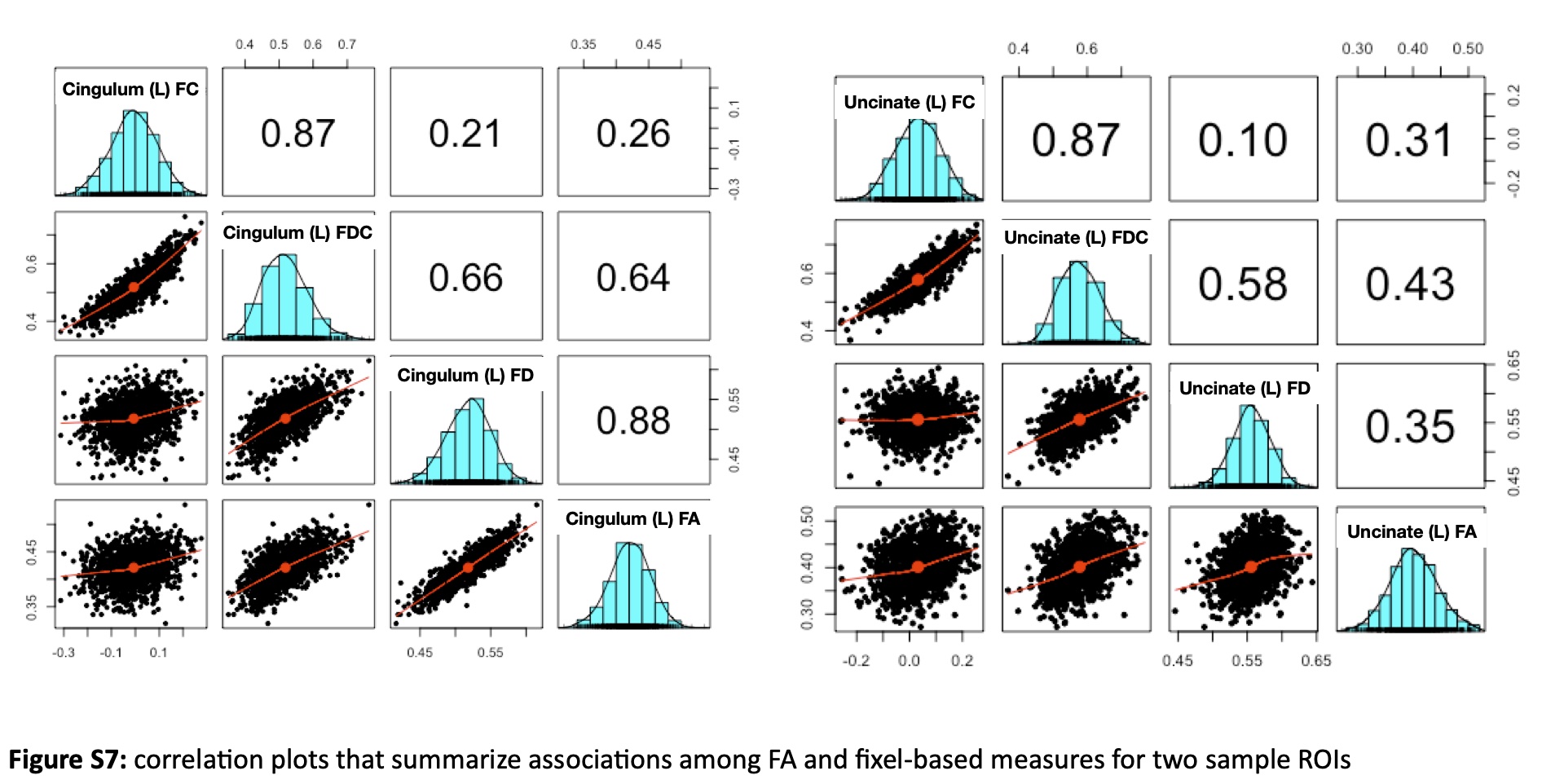

Supplement: Supplementary file 10 — Figure S7 [file 41398_2022_2013_MOESM10_ESM.jpg]
