# Supplementary material for: Obsessive compulsive symptom dimensions are linked to altered white-matter microstructure in a community sample of youth
Source: Transl Psychiatry. 2022 Aug 10;12:328. doi: 10.1038/s41398-022-02013-w (PMC9365814; doi:10.1038/s41398-022-02013-w)
Supplement: Supplementary file 11 — Supplemental Materials – Table of Contents [file 41398_2022_2013_MOESM11_ESM.docx]

**Supplemental Materials – Table of Contents**

**Table S1:** Sample characteristics for the overall sample (N=8,898)

**Table S2a:** Factor Analysis: Obsessive Compulsive Symptom 4-factor model

**Table S2b:** Factor Analysis: P-factor Model

**Table S2c:** Factor Analysis: OCS Single factor model

**Tables S3a-l**: Fixel-based a priori ROI results

**Tables S4a-l:** Voxel-based a priori ROI results

**Table S5:** exploratory models for nonlinear age covariate and age-interaction effects

**Table S6:** exploratory models for *g*-factor (IQ) effects

**Table S7:** exploratory models for age effects

**Figure S1:** Voxel-based representation of fixel-based and voxel-based ROIs

**Figure S2**: Alternative representation of the fixel-based results. Tractography for each significant region was performed and coded with a separate color. This may aid the reader in understanding the extent to which the ROIs overlap

**Figure S3:** exploratory results when a quadratic age term was incorporated into our whole-brain model for fixel-based analysis

**Figure S4a:** exploratory results for age effects (fixel-based)

**Figure S4b:** exploratory results for age effects (tensor/FA-based)

**Figure S5a:** exploratory results for *g*-factor (IQ) effects (fixel-based)

**Figure S5b:** exploratory results for *g*-factor (IQ) effects (tensor/FA-based)

**Figure S6:** correlation plots that summarize associations between OCS scores, p-factor scores, cognition, and maternal years of education

**Figure S7:** correlation plots that summarize associations among FA and fixel-based measures for two sample ROIs

## Table S1. Demographics– Overall Sample

N=8,898*

| **Variable** |  |  |
| --- | --- | --- |
| Sex |  |  |
| %Male | 48.0% |  |
| %Female | 52.0% |  |
| Mean Age (SD) | 13.53 (3.58) |  |
| Maternal Education  in Years (SD) | 14.48 (2.43) |  |
| Ethnicity |  |  |
| % White | 55.07% |  |
| % Black | 32.88% |  |
| % Other | 12.05% |  |

## *Subset of the 9,496 with non-missing data on the 12 OCS items.

## S2A. Factor Analysis Table – Obsessive Compulsive Symptoms

*Results From a Factor Analysis of 16 Obsessive Compulsive Items* from the GOASSESS*

| GOASSESS OCD item | Factor loading | | | |
| --- | --- | --- | --- | --- |
|  | 1 | 2 | 3 | 4 |
| **Factor 1 - Bad Thoughts** |  |  |  |  |
| 001. Have you ever been bothered by thoughts that don't make sense to you, that come over and over again and won't go away, such as concern with harming others/self? | **0.866** |  |  |  |
| 005. Have you ever been bothered by thoughts that don't make sense to you, that come over and over again and won't go away, such as feelings that bad things that happened were your fault? | **0.776** |  |  |  |
| 004 Have you ever been bothered by thoughts that don't make sense to you, that come over and over again and won't go away, such as fear that you would do something/say something bad without intending to? | **0.783** | 0.153 |  |  |
| 006. Have you ever been bothered by thoughts that don't make sense to you, that come over and over again and won't go away, such as forbidden/bad thoughts? | **0.884** |  |  |  |
| 008. Have you ever been bothered by thoughts that don't make sense to you, that come over and over again and won't go away, such as religious thoughts? | **0.555** |  |  | 0.209 |
| 002. Have you ever been bothered by thoughts that don't make sense to you, that come over and over again and won't go away, such as pictures of violent things? | **0.795** |  |  |  |
| **Factor 2 - Symmetry** |  |  |  |  |
| 007. Have you ever been bothered by thoughts that don't make sense to you, that come over and over again and won't go away, such as need for symmetry/exactness? |  | **0.889** | -0.102 |  |
| 016. Have you ever had to do something over and over again - that would have made you feel really nervous if you couldn't do it, like: ordering or arranging things? |  | **0.749** | 0.255 |  |
| 019. Do you feel the need to do things just right (like they have to be perfect)? |  | **0.665** |  |  |
| **Factor 3 - Repetition/Checking** |  |  |  |  |
| 017. Have you ever had to do something over and over again - that would have made you feel really nervous if you couldn't do it, like: doing things over and over again at bedtime, like arranging the pillows, sheets, or other things? |  | 0.29 | **0.548** |  |
| 014. Have you ever had to do something over and over again - that would have made you feel really nervous if you couldn't do it, like: getting dressed over and over again? |  |  | **0.711** | 0.148 |
| 015. Have you ever had to do something over and over again - that would have made you feel really nervous if you couldn't do it, like: going in and out a door over and over again? | 0.143 |  | **0.779** |  |
| **Factor 4 - Contamination** |  |  |  |  |
| 003. Have you ever been bothered by thoughts that don't make sense to you, that come over and over again and won't go away, such as thoughts about contamination/germs/illness? | 0.102 |  |  | **0.899** |
| 011. Have you ever had to do something over and over again - that would have made you feel really nervous if you couldn't do it, like: cleaning or washing (for example, your hands, house)? |  |  | 0.328 | **0.653** |
| 013. Have you ever had to do something over and over again - that would have made you feel really nervous if you couldn't do it, like: checking (for example, doors, locks, ovens)? | 0.174 | 0.192 | **0.423** | 0.167 |
| 012. Have you ever had to do something over and over again - that would have made you feel really nervous if you couldn't do it, like: counting? |  | **0.361** | **0.365** | 0.107 |

*Note. N* = 8,898. The extraction method was principal axis factoring with an oblique (Oblimin) rotation. Factor loadings above .30 are in bold; factor loadings below .10 are omitted.

## S2B. Factor Analysis Table – P-factor model

| **GOASSESS Item** | **Item Loading** |
| --- | --- |
| DEP004. Depression: Has there ever been a time when you felt grouchy, irritable or in a bad mood most of the time; even little things would make you mad? | 0.715 |
| MAN007. Mania/ Hypomania: Has there ever been a time when you felt unusually grouchy, cranky, or irritable; when the smallest things would make you really mad? | 0.708 |
| SIP014. SIPS- PRIME SCREEN-REVISED Structured Interview for Prodromal Symptoms: I have been concerned that I might be "going crazy." | 0.705 |
| MAN004. Mania/ Hypomania: Have there been times when you kept talking a lot, couldn't stop talking, talked faster than usual, had thoughts faster than usual, or had so many ideas in your head that you could hardly keep track of them? | 0.7 |
| DEP006. Depression: Has there ever been a time when nothing was fun for you and you just weren't interested in anything? | 0.695 |
| ODD006. Oppositional Defiant Disorder: Were you often irritable or grouchy, or did you often get angry because you thought that things were unfair? | 0.691 |
| MAN001. Mania/ Hypomania: Have there been times when you were much more active, excited or energetic than usual, had problems sitting still, or needed to move around a lot? | 0.69 |
| MAN002. Mania/ Hypomania: Has there ever been a time when you felt so full of energy that you couldn't stop doing things and didn't get tired? | 0.686 |
| MAN005. Mania/ Hypomania: Have you ever had a time when you felt much more happy or excited than you usually do when there was nothing special going on? | 0.678 |
| SIP012. SIPS- PRIME SCREEN-REVISED Structured Interview for Prodromal Symptoms: I have had the experience of hearing faint or clear sounds of people or a person mumbling or talking when there is no one near me. | 0.673 |
| MAN003. Mania/ Hypomania: Has there ever been a time when you felt like you hardly needed sleep? | 0.669 |
| SIP011. SIPS- PRIME SCREEN-REVISED Structured Interview for Prodromal Symptoms: I think I might feel like my mind is "playing tricks" on me. | 0.655 |
| PAN004. Panic Disorder: Has there ever been a time when all of a sudden, you felt that you were losing control, something terrible was going to happen, that you were going crazy, or going to die? | 0.654 |
| SIP007. SIPS- PRIME SCREEN-REVISED Structured Interview for Prodromal Symptoms: I think I may get confused at times whether something I experience or perceive may be real or may be just part of my imagination or dreams. | 0.649 |
| PSY060. Psychosis: Have you ever had strange feelings in your body like things were crawling on you or someone touching you and nothing or no one was there? | 0.642 |
| SIP009. SIPS- PRIME SCREEN-REVISED Structured Interview for Prodromal Symptoms: I wonder if people may be planning to hurt me or even may be about to hurt me. | 0.639 |
| SIP003. SIPS- PRIME SCREEN-REVISED: I think that I have felt that there are odd or unusual things going on that I can't explain. | 0.637 |
| PSY001. Psychosis: Have you ever heard voices when no one was there? | 0.636 |
| DEP001. Depression: Has there ever been a time when you felt sad or depressed most of the time? | 0.633 |
| ODD002. Oppositional Defiant Disorder: Was there a time when you often got into trouble with adults for refusing to do what they told you to do or for breaking rules at home/school? | 0.631 |
| CDD008. Conduct Disorder: Did you ever: threaten someone? | 0.628 |
| DEP002. Depression: Has there ever been a time when you cried a lot, or felt like crying? | 0.622 |
| ODD001. Oppositional Defiant Disorder: Was there a time when you often did things that got you into trouble with adults such as losing your temper, arguing with or talking back to adults, or being grouchy or irritable with them? | 0.62 |
| ADD016. Attention Deficit Disorder: Did you often have people tell you that you did not seem to be listening when they spoke to you or that you were daydreaming? | 0.62 |
| MAN006. Mania/ Hypomania: Have you ever had a time when you felt like you could do almost anything? | 0.616 |
| PSY071. Psychosis: Have you ever believed in things and later found out they weren't true, like people being out to get you, or talking about you behind your back, or controlling what you do or think? | 0.616 |
| SIP013. SIPS- PRIME SCREEN-REVISED Structured Interview for Prodromal Symptoms: I think that I may hear my own thoughts being said out loud. | 0.612 |
| SUI002. Suicide: Have you ever thought about killing yourself? | 0.603 |
| SIP032. SIPS- Structured Interview for Prodromal Symptoms: Do you ever feel a loss of sense of self or feel disconnected from yourself or your life? | 0.603 |
| PSY029. Psychosis: Have you ever seen visions or seen things which other people could not see? | 0.595 |
| SIP005. SIPS- PRIME SCREEN-REVISED- Structured Interview for Prodromal Symptoms: I may have felt that there could possibly be something interrupting or controlling my thoughts, feelings, or actions. | 0.593 |
| AGR008. Agoraphobia: Looking at this card, have you ever been very nervous or afraid of: using public transportation like a bus or SEPTA? | 0.59 |
| PAN003. Panic Disorder: Has there ever been a time when all of a sudden you felt very, very scared or uncomfortable - and your chest hurt, you couldn't catch your breath, your heart beat very fast, you felt very shaky, and sweaty/tingly/numb in your hands or feet? | 0.587 |
| AGR001. Agoraphobia: Looking at this card, have you ever been very nervous or afraid of: being in crowds (for example, a classroom, cafeteria, restaurant, or movie theater)? | 0.582 |
| AGR002. Agoraphobia: Looking at this card, have you ever been very nervous or afraid of: going to public places (such as a store or shopping mall)? | 0.581 |
| ADD011. Attention Deficit Disorder: Did you often have trouble paying attention or keeping your mind on your school, work, chores, or other activities that you were doing? | 0.58 |
| SIP006. SIPS- PRIME SCREEN-REVISED- Structured Interview for Prodromal Symptoms: I have had the experience of doing something differently because of my superstitions. | 0.578 |
| ADD020. Attention Deficit Disorder: Did you often have difficulty sitting still for more than a few minutes at a time, even after being asked to stay seated, or did you often fidget with your hands or feet or wiggle in your seat or were you "always on the go"? | 0.577 |
| PSY020. Psychosis: Did you ever hear other sounds or noises that other people couldn't hear? | 0.576 |
| ODD005. Oppositional Defiant Disorder: Did you ever get into trouble for getting even with other people by doing things to hurt them, telling lies about them, or messing up their things? | 0.575 |
| SUI001. Suicide: Have you ever thought a lot about death or dying? | 0.571 |
| CDD005. Conduct Disorder: Did you often bully others (hitting, threatening or scaring someone who was younger or smaller), threaten or frighten someone on purpose, or often start physical fights with others? | 0.571 |
| PSY070. Psychosis: Have you ever believed in things that most other people or your parents don't believe in? | 0.569 |
| AGR006. Agoraphobia: Looking at this card, have you ever been very nervous or afraid of: traveling away from home? | 0.569 |
| SIP033. SIPS- Structured Interview for Prodromal Symptoms: Has anyone pointed out to you that you are less emotional or connected to people than you used to be? | 0.568 |
| ADD015. Attention Deficit Disorder: Did you often have trouble making plans, doing things that had to be done in a certain kind of order, or that had a lot of different steps? | 0.563 |
| CDD007. Conduct Disorder: Did you ever: try to hurt someone with a weapon (a bat, brick, broken bottle, knife, or gun)? | 0.558 |
| ODD003. Oppositional Defiant Disorder: Did you often annoy other people on purpose or blame other people for your mistakes (excluding siblings)? | 0.558 |
| CDD003. Conduct Disorder: Did you ever set fires, break into cars, or destroy someone else's property on purpose? | 0.55 |
| ADD013. Attention Deficit Disorder: Did you often dislike, avoid, or put off school or homework (or any other activity requiring concentration) | 0.547 |
| ADD012. Attention Deficit Disorder: Did you often have problems following instructions and often fail to finish school, work, or other things you meant to get done? | 0.545 |
| CDD006. Conduct Disorder: Have you ever been physically cruel to an animal or person (on purpose)? | 0.54 |
| AGR003. Agoraphobia: Looking at this card, have you ever been very nervous or afraid of: being in an open field? | 0.54 |
| CDD001. Conduct Disorder: Was there ever a time when you often did things that got you into trouble with adults like lying or stealing (something worth more than $5, from family, others, or stores)? | 0.538 |
| PSY050. Psychosis: Have you ever smelled strange odors other people could not smell? | 0.537 |
| AGR004. Agoraphobia: Looking at this card, have you ever been very nervous or afraid of: going over bridges or through tunnels? | 0.531 |
| ADD014. Attention Deficit Disorder: Did you often lose things you needed for school or projects at home (assignments or books) or make careless mistakes in school work or other activities? | 0.528 |
| SIP008. SIPS- PRIME SCREEN-REVISED Structured Interview for Prodromal Symptoms: I have thought that it might be possible that other people can read my mind, or that I can read others' minds | 0.526 |
| ADD022. Attention Deficit Disorder: Did you often join other people's conversations or have trouble waiting your turn (e.g., waiting in line, waiting for a teacher to call on you in class)? | 0.524 |
| CDD009. Conduct Disorder: Did you ever: hold someone up? | 0.518 |
| AGR005. Agoraphobia: Looking at this card, have you ever been very nervous or afraid of: traveling by yourself? | 0.517 |
| SIP010. SIPS- PRIME SCREEN-REVISED Structured Interview for Prodromal Symptoms: I believe that I have special natural or supernatural gifts beyond my talents and natural strengths. | 0.516 |
| SOC005. Social Anxiety: Looking at this card, was there ever a time in your life when you felt afraid or uncomfortable because you were the center of attention and were concerned something embarrassing might happen and you felt very afraid or felt uncomfortable? | 0.512 |
| SEP510. Separation Anxiety: Did you ever worry/have bad dreams about something terrible happening to you or your (attachment figures) so that you would not see them again? | 0.51 |
| PAN001. Panic Disorder: Have you ever had an attack like this? | 0.501 |
| SIP028. SIPS- Structured Interview for Prodromal Symptoms: Do people ever seem to have difficulty understanding you? | 0.501 |
| ADD021. Attention Deficit Disorder: Did you often blurt out answers to other people's questions before they finished speaking or interrupt people abruptly? | 0.499 |
| CDD002. Conduct Disorder: Did you ever skip school, stay out at night later than you were supposed to (more than 2 hours), or run away from home overnight? | 0.498 |
| SCR007. General Probes: Have you ever had to go to a hospital and stay overnight because of problems with your mood, feelings, or how you were acting? | 0.493 |
| SCR001. General Probes: Have you ever talked to a counselor, psychologist, social worker, psychiatrist or some other professional about your feelings or problems with your mood or behaviors? | 0.489 |
| SIP027. SIPS- Structured Interview for Prodromal Symptoms: Do people ever tell you that they can't understand you? | 0.483 |
| SOC004. Social Anxiety: Looking at this card, was there ever a time in your life when you felt afraid or uncomfortable acting, performing, giving a talk/speech, playing a sport or doing a musical performance, or taking an important test or exam (even though you studied enough)? | 0.482 |
| SOC001. Social Anxiety: Looking at this card, was there ever a time in your life when you felt afraid or uncomfortable or really, really shy with people, like meeting new people, going to parties, or eating or drinking, writing or doing homework in front of others? | 0.476 |
| SIP004. SIPS- PRIME SCREEN-REVISED: I think that I might be able to predict the future. | 0.475 |
| CDD010. Conduct Disorder: Did you ever: attack someone to steal from them? | 0.466 |
| GAD002. Generalized Anxiety Disorder: Did you worry a lot more than most children/people your age? | 0.465 |
| AGR007. Agoraphobia: Looking at this card, have you ever been very nervous or afraid of: traveling in a car? | 0.462 |
| SIP039. SIPS- Structured Interview for Prodromal Symptoms: Within the past 6 months, are you having a harder time getting normal activities done? | 0.446 |
| SOC002. Social Anxiety: Looking at this card, was there ever a time in your life when you felt afraid or uncomfortable talking on the telephone or with people your own age who you don't know very well? | 0.445 |
| PHB007. Specific Phobia: Looking at this card, have you ever been very nervous or afraid of flying or airplanes? | 0.428 |
| GAD001. Generalized Anxiety Disorder: Have you ever been a worrier? | 0.427 |
| PHB006. Specific Phobia: Looking at this card, have you ever been very nervous or afraid of closed spaces, like elevators or closets? | 0.427 |
| SCR006. General Probes: Are you currently taking medication because of your emotions and/or behaviors? | 0.419 |
| SEP500. Separation Anxiety: Since you were 5 years old, has there ever been a time when you had a lot of worries about your (attachment figures) and were very upset or got sick (for example, felt sick to your stomach, headaches, thrown-up) when you were away from him/her? | 0.418 |
| SOC003. Social Anxiety: Looking at this card, was there ever a time in your life when you felt afraid or uncomfortable when you had to do something in front of a group of people, like speaking in class? | 0.415 |
| CDD011. Conduct Disorder: Did you ever: trick or threaten someone into having sex with you, or did anyone ever accuse you of making them do something sexual? | 0.412 |
| PHB003. Specific Phobia: Looking at this card, have you ever been very nervous or afraid of water or situations involving water, such as a swimming pool, lake, or ocean? | 0.4 |
| SIP038. SIPS- Structured Interview for Prodromal Symptoms: Within the past 6 months, are you having a harder time getting your work or schoolwork done? | 0.398 |
| SEP509. Separation Anxiety: When you knew that you were going to be away from home or (attachment figure(s)), did you get very upset and worry (e.g., when you learned (attachment figure(s)) were going on an upcoming trip or night out)? | 0.398 |
| PHB002. Specific Phobia: Looking at this card, have you ever been very nervous or afraid of being in really high places, like a roof or tall building? | 0.375 |
| SEP508. Separation Anxiety: Has there ever been a time when you wanted to stay home from school or not go to other places (for example, sleep-overs) without your (attachment figures)? | 0.374 |
| PHB008. Specific Phobia: Looking at this card, have you ever been very nervous or afraid of any other things or situations? | 0.366 |
| CDD004. Conduct Disorder: Do you have a probation officer or have you ever been on probation? | 0.362 |
| PHB001. Specific Phobia: Looking at this card, have you ever been very nervous or afraid of animals or bugs, like dogs, snakes, or spiders? | 0.311 |
| PHB004. Specific Phobia: Looking at this card, have you ever been very nervous or afraid of storms, thunder, or lightning? | 0.306 |
| SEP511. Separation Anxiety: Were you scared to be alone in your room (or any place in your house) or did you need your (attachment figure(s)) to stay with you while you fell asleep? | 0.268 |
| PHB005. Specific Phobia: Looking at this card, have you ever been very nervous or afraid of doctors, needles, or blood? | 0.225 |

## S2B. Factor Analysis Table – P-factor model item loadings

## S2C. Factor Analysis Table – Obsessive Compulsive Single Factor model item loadings

**Tables S3a-l: Fixel-based ROI analyses**

| **Model: <ROI> <DV> ~ P-factor + Covariates** | *B* | *p_orig_* | *p_FDR_* |
| --- | --- | --- | --- |
| ROI: Cingulum Left |  |  |  |
| FC | 0.003 | 0.944 | 0.990 |
| FDC | -0.013 | 0.749 | 0.990 |
| FD | -0.031 | 0.437 | 0.990 |
| ROI: Cingulum Right |  |  |  |
| FC | 0.000 | 0.990 | 0.990 |
| FDC | 0.000 | 0.990 | 0.990 |
| FD | 0.005 | 0.906 | 0.990 |
| ROI: Callosum Body |  |  |  |
| FC | 0.003 | 0.946 | 0.990 |
| FDC | 0.019 | 0.625 | 0.990 |
| FD | 0.050 | 0.237 | 0.990 |
| ROI: Callosum Genu |  |  |  |
| FC | 0.015 | 0.748 | 0.990 |
| FDC | 0.016 | 0.716 | 0.990 |
| FD | 0.018 | 0.641 | 0.990 |
| ROI: Callosum Splenium |  |  |  |
| FC | -0.008 | 0.860 | 0.990 |
| FDC | -0.006 | 0.889 | 0.990 |
| FD | 0.008 | 0.861 | 0.990 |
| ROI: OFC Left |  |  |  |
| FC | -0.023 | 0.593 | 0.990 |
| FDC | -0.005 | 0.911 | 0.990 |
| FD | 0.020 | 0.661 | 0.990 |
| ROI: OFC Right |  |  |  |
| FC | -0.016 | 0.700 | 0.990 |
| FDC | -0.007 | 0.866 | 0.990 |
| FD | 0.005 | 0.911 | 0.990 |
| ROI: PTR Left |  |  |  |
| FC | 0.002 | 0.969 | 0.990 |
| FDC | 0.015 | 0.706 | 0.990 |
| FD | 0.027 | 0.555 | 0.990 |
| ROI: PTR Right |  |  |  |
| FC | -0.012 | 0.779 | 0.990 |
| FDC | -0.045 | 0.269 | 0.990 |
| FD | -0.060 | 0.190 | 0.990 |
| ROI: Saggital Striatum Left |  |  |  |
| FC | 0.003 | 0.931 | 0.990 |
| FDC | -0.015 | 0.715 | 0.990 |
| FD | -0.027 | 0.544 | 0.990 |
| ROI: Saggital Striatum Right |  |  |  |
| FC | 0.024 | 0.543 | 0.990 |
| FDC | 0.006 | 0.875 | 0.990 |
| FD | -0.024 | 0.598 | 0.990 |
| ROI: Uncinate Left |  |  |  |
| FC | 0.017 | 0.673 | 0.990 |
| FDC | -0.007 | 0.862 | 0.990 |
| FC | -0.034 | 0.453 | 0.990 |
| ROI: Uncinate Right |  |  |  |
| FC | 0.019 | 0.633 | 0.990 |
| FDC | 0.045 | 0.269 | 0.990 |
| FC | 0.049 | 0.260 | 0.990 |
| ROI: SCS Left |  |  |  |
| FC | -0.032 | 0.383 | 0.990 |
| FDC | -0.027 | 0.469 | 0.990 |
| FC | 0.001 | 0.974 | 0.990 |

**Supplemental table 3a:** standardized betas and p-values for the models testing the association between P-factor scores and the 14 fixel ROIs. The full model with covariates can be represented as ROI ~ P-factor + Bad Thoughts + Repetition/Checking + Symmetry + Contamination + g + age + gender + race + QualityMetric. SCS = Superior Corticostriatial, PTR = Posterior Thalamic Radiation, OFC = Orbitofrontal Cortex

| **Model: <ROI> <DV> ~ Repetition/Checking + Covariates** | *B* | *p_orig_* | *p_FDR_* |
| --- | --- | --- | --- |
| ROI: Cingulum Left |  |  |  |
| FC | -0.014 | 0.741 | 0.921 |
| FDC | -0.028 | 0.496 | 0.760 |
| FD | -0.029 | 0.497 | 0.760 |
| ROI: Cingulum Right |  |  |  |
| FC | -0.002 | 0.958 | 0.963 |
| FDC | -0.015 | 0.728 | 0.921 |
| FD | -0.029 | 0.507 | 0.760 |
| ROI: Callosum Body |  |  |  |
| FC | 0.038 | 0.367 | 0.705 |
| FDC | 0.007 | 0.871 | 0.963 |
| FD | -0.045 | 0.313 | 0.705 |
| ROI: Callosum Genu |  |  |  |
| FC | 0.022 | 0.650 | 0.881 |
| FDC | 0.002 | 0.963 | 0.963 |
| FD | -0.035 | 0.390 | 0.712 |
| ROI: Callosum Splenium |  |  |  |
| FC | 0.074 | 0.101 | 0.463 |
| FDC | 0.054 | 0.210 | 0.640 |
| FD | -0.058 | 0.213 | 0.640 |
| ROI: OFC Left |  |  |  |
| FC | 0.075 | 0.094 | 0.463 |
| FDC | 0.080 | 0.069 | 0.463 |
| FD | 0.025 | 0.601 | 0.849 |
| ROI: OFC Right |  |  |  |
| FC | 0.041 | 0.345 | 0.705 |
| FDC | 0.032 | 0.473 | 0.760 |
| FD | -0.009 | 0.859 | 0.963 |
| ROI: PTR Left |  |  |  |
| FC | 0.103 | 0.018 | 0.385 |
| FDC | 0.069 | 0.108 | 0.463 |
| FD | -0.013 | 0.786 | 0.921 |
| ROI: PTR Right |  |  |  |
| FC | 0.112 | 0.011 | 0.385 |
| FDC | 0.089 | 0.039 | 0.406 |
| FD | 0.002 | 0.959 | 0.963 |
| ROI: Saggital Striatum Left |  |  |  |
| FC | 0.030 | 0.470 | 0.760 |
| FDC | 0.057 | 0.178 | 0.625 |
| FD | 0.054 | 0.260 | 0.705 |
| ROI: Saggital Striatum Right |  |  |  |
| FC | 0.041 | 0.321 | 0.705 |
| FDC | 0.022 | 0.606 | 0.849 |
| FD | -0.014 | 0.771 | 0.921 |
| ROI: Uncinate Left |  |  |  |
| FC | -0.003 | 0.940 | 0.963 |
| FDC | -0.049 | 0.269 | 0.705 |
| FC | -0.065 | 0.165 | 0.625 |
| ROI: Uncinate Right |  |  |  |
| FC | -0.011 | 0.790 | 0.921 |
| FDC | -0.069 | 0.110 | 0.463 |
| FC | -0.101 | 0.030 | 0.406 |
| ROI: SCS Left |  |  |  |
| FC | 0.069 | 0.074 | 0.463 |
| FDC | 0.041 | 0.295 | 0.705 |
| FC | -0.042 | 0.369 | 0.705 |

**Supplemental table 3b:** standardized betas and p-values for the models testing the association between Repetition/Checking and the 14 fixel ROIs. The full model with covariates can be represented as ROI ~ P-factor + Bad Thoughts + Repetition/Checking + Symmetry + Contamination + g + age + gender + race + QualityMetric. SCS = Superior Corticostriatial, PTR = Posterior Thalamic Radiation, OFC = Orbitofrontal Cortex

| **Model: <ROI> <DV> ~ Bad Thoughts + Covariates** | *B* | *p_orig_* | *p_FDR_* |
| --- | --- | --- | --- |
| ROI: Cingulum Left |  |  |  |
| FC | 0.067 | 0.085 | 0.359 |
| FDC | 0.078 | 0.048 | 0.332 |
| FD | 0.056 | 0.164 | 0.598 |
| ROI: Cingulum Right |  |  |  |
| FC | 0.072 | 0.071 | 0.332 |
| FDC | 0.045 | 0.261 | 0.684 |
| FD | -0.018 | 0.655 | 0.880 |
| ROI: Callosum Body |  |  |  |
| FC | 0.000 | 0.994 | 0.994 |
| FDC | 0.043 | 0.260 | 0.684 |
| FD | 0.081 | 0.056 | 0.332 |
| ROI: Callosum Genu |  |  |  |
| FC | 0.013 | 0.775 | 0.880 |
| FDC | 0.041 | 0.358 | 0.801 |
| FD | 0.071 | 0.070 | 0.332 |
| ROI: Callosum Splenium |  |  |  |
| FC | 0.010 | 0.812 | 0.897 |
| FDC | 0.052 | 0.212 | 0.654 |
| FD | 0.111 | 0.012 | 0.332 |
| ROI: OFC Left |  |  |  |
| FC | -0.004 | 0.930 | 0.976 |
| FDC | 0.013 | 0.750 | 0.880 |
| FD | 0.034 | 0.454 | 0.865 |
| ROI: OFC Right |  |  |  |
| FC | 0.029 | 0.491 | 0.865 |
| FDC | 0.032 | 0.452 | 0.865 |
| FD | 0.024 | 0.606 | 0.865 |
| ROI: PTR Left |  |  |  |
| FC | 0.013 | 0.763 | 0.880 |
| FDC | 0.021 | 0.611 | 0.865 |
| FD | 0.017 | 0.706 | 0.880 |
| ROI: PTR Right |  |  |  |
| FC | 0.051 | 0.218 | 0.654 |
| FDC | 0.091 | 0.027 | 0.332 |
| FD | 0.092 | 0.045 | 0.332 |
| ROI: Saggital Striatum Left |  |  |  |
| FC | 0.042 | 0.297 | 0.733 |
| FDC | 0.022 | 0.589 | 0.865 |
| FD | -0.023 | 0.618 | 0.865 |
| ROI: Saggital Striatum Right |  |  |  |
| FC | 0.024 | 0.552 | 0.865 |
| FDC | 0.005 | 0.898 | 0.967 |
| FD | -0.030 | 0.512 | 0.865 |
| ROI: Uncinate Left |  |  |  |
| FC | 0.029 | 0.476 | 0.865 |
| FDC | 0.013 | 0.750 | 0.880 |
| FC | -0.001 | 0.987 | 0.994 |
| ROI: Uncinate Right |  |  |  |
| FC | 0.024 | 0.548 | 0.865 |
| FDC | -0.013 | 0.749 | 0.880 |
| FC | -0.040 | 0.362 | 0.801 |
| ROI: SCS Left |  |  |  |
| FC | 0.050 | 0.171 | 0.598 |
| FDC | 0.086 | 0.019 | 0.332 |
| FC | 0.094 | 0.032 | 0.332 |

**Supplemental table 3c:** standardized betas and p-values for the models testing the association between Bad Thoughts scores and the 14 fixel ROIs. The full model with covariates can be represented as ROI ~ P-factor + Bad Thoughts + Repetition/Checking + Symmetry + Contamination + g + age + gender + race + QualityMetric. SCS = Superior Corticostriatial, PTR = Posterior Thalamic Radiation, OFC = Orbitofrontal Cortex

| **Model: <ROI> <DV> ~ Symmetry + Covariates** | *B* | *p_orig_* | *p_FDR_* |
| --- | --- | --- | --- |
| ROI: Cingulum Left |  |  |  |
| FC | -0.058 | 0.123 | 0.399 |
| FDC | -0.067 | 0.080 | 0.372 |
| FD | -0.051 | 0.190 | 0.399 |
| ROI: Cingulum Right |  |  |  |
| FC | -0.052 | 0.169 | 0.399 |
| FDC | -0.051 | 0.189 | 0.399 |
| FD | -0.013 | 0.746 | 0.895 |
| ROI: Callosum Body |  |  |  |
| FC | -0.027 | 0.486 | 0.731 |
| FDC | -0.051 | 0.170 | 0.399 |
| FD | -0.060 | 0.139 | 0.399 |
| ROI: Callosum Genu |  |  |  |
| FC | -0.083 | 0.059 | 0.372 |
| FDC | -0.076 | 0.074 | 0.372 |
| FD | -0.015 | 0.693 | 0.859 |
| ROI: Callosum Splenium |  |  |  |
| FC | -0.038 | 0.357 | 0.625 |
| FDC | -0.071 | 0.074 | 0.372 |
| FD | -0.089 | 0.035 | 0.372 |
| ROI: OFC Left |  |  |  |
| FC | -0.042 | 0.301 | 0.550 |
| FDC | -0.066 | 0.102 | 0.399 |
| FD | -0.045 | 0.296 | 0.550 |
| ROI: OFC Right |  |  |  |
| FC | -0.055 | 0.173 | 0.399 |
| FDC | -0.057 | 0.156 | 0.399 |
| FD | -0.011 | 0.803 | 0.924 |
| ROI: PTR Left |  |  |  |
| FC | -0.052 | 0.190 | 0.399 |
| FDC | -0.050 | 0.202 | 0.404 |
| FD | -0.017 | 0.696 | 0.859 |
| ROI: PTR Right |  |  |  |
| FC | -0.058 | 0.149 | 0.399 |
| FDC | -0.035 | 0.377 | 0.634 |
| FD | 0.020 | 0.650 | 0.853 |
| ROI: Saggital Striatum Left |  |  |  |
| FC | -0.078 | 0.042 | 0.372 |
| FDC | -0.082 | 0.036 | 0.372 |
| FD | -0.030 | 0.495 | 0.731 |
| ROI: Saggital Striatum Right |  |  |  |
| FC | -0.031 | 0.408 | 0.659 |
| FDC | -0.026 | 0.505 | 0.731 |
| FD | 0.009 | 0.836 | 0.924 |
| ROI: Uncinate Left |  |  |  |
| FC | -0.004 | 0.909 | 0.933 |
| FDC | -0.009 | 0.831 | 0.924 |
| FC | -0.007 | 0.872 | 0.933 |
| ROI: Uncinate Right |  |  |  |
| FC | 0.017 | 0.649 | 0.853 |
| FDC | -0.001 | 0.971 | 0.971 |
| FC | -0.005 | 0.911 | 0.933 |
| ROI: SCS Left |  |  |  |
| FC | -0.022 | 0.540 | 0.756 |
| FDC | -0.063 | 0.076 | 0.372 |
| FC | -0.091 | 0.031 | 0.372 |

**Supplemental table 3d:** standardized betas and p-values for the models testing the association between Symmetry Scores and the 14 fixel ROIs. The full model with covariates can be represented as ROI ~ P-factor + Bad Thoughts + Repetition/Checking + Symmetry + Contamination + g + age + gender + race + QualityMetric. SCS = Superior Corticostriatial, PTR = Posterior Thalamic Radiation, OFC = Orbitofrontal Cortex

| **Model: <ROI> <DV> ~ Contamination + Covariates** | *B* | *p_orig_* | *p_FDR_* |
| --- | --- | --- | --- |
| ROI: Cingulum Left |  |  |  |
| FC | -0.014 | 0.716 | 0.961 |
| FDC | 0.005 | 0.889 | 0.961 |
| FD | 0.026 | 0.506 | 0.961 |
| ROI: Cingulum Right |  |  |  |
| FC | -0.010 | 0.787 | 0.961 |
| FDC | 0.010 | 0.802 | 0.961 |
| FD | 0.023 | 0.557 | 0.961 |
| ROI: Callosum Body |  |  |  |
| FC | -0.017 | 0.672 | 0.961 |
| FDC | -0.030 | 0.418 | 0.961 |
| FD | -0.035 | 0.397 | 0.961 |
| ROI: Callosum Genu |  |  |  |
| FC | 0.023 | 0.602 | 0.961 |
| FDC | 0.005 | 0.906 | 0.961 |
| FD | -0.041 | 0.276 | 0.961 |
| ROI: Callosum Splenium |  |  |  |
| FC | 0.002 | 0.959 | 0.969 |
| FDC | -0.004 | 0.911 | 0.961 |
| FD | 0.005 | 0.911 | 0.961 |
| ROI: OFC Left |  |  |  |
| FC | -0.015 | 0.715 | 0.961 |
| FDC | -0.025 | 0.535 | 0.961 |
| FD | -0.023 | 0.597 | 0.961 |
| ROI: OFC Right |  |  |  |
| FC | -0.016 | 0.685 | 0.961 |
| FDC | -0.010 | 0.799 | 0.961 |
| FD | 0.002 | 0.969 | 0.969 |
| ROI: PTR Left |  |  |  |
| FC | -0.035 | 0.389 | 0.961 |
| FDC | -0.044 | 0.265 | 0.961 |
| FD | -0.024 | 0.583 | 0.961 |
| ROI: PTR Right |  |  |  |
| FC | -0.055 | 0.174 | 0.961 |
| FDC | -0.090 | 0.024 | 0.511 |
| FD | -0.095 | 0.032 | 0.511 |
| ROI: Saggital Striatum Left |  |  |  |
| FC | -0.018 | 0.650 | 0.961 |
| FDC | 0.007 | 0.864 | 0.961 |
| FD | 0.035 | 0.423 | 0.961 |
| ROI: Saggital Striatum Right |  |  |  |
| FC | -0.030 | 0.434 | 0.961 |
| FDC | -0.028 | 0.478 | 0.961 |
| FD | -0.012 | 0.794 | 0.961 |
| ROI: Uncinate Left |  |  |  |
| FC | -0.014 | 0.725 | 0.961 |
| FDC | 0.054 | 0.180 | 0.961 |
| FC | 0.091 | 0.037 | 0.511 |
| ROI: Uncinate Right |  |  |  |
| FC | -0.039 | 0.315 | 0.961 |
| FDC | 0.004 | 0.916 | 0.961 |
| FC | 0.034 | 0.424 | 0.961 |
| ROI: SCS Left |  |  |  |
| FC | -0.061 | 0.085 | 0.893 |
| FDC | -0.042 | 0.243 | 0.961 |
| FC | 0.018 | 0.677 | 0.961 |

**Supplemental table 3e:** standardized betas and p-values for the models testing the association between Contamination Scores and the 14 fixel ROIs. The full model with covariates can be represented as ROI ~ P-factor + Bad Thoughts + Repetition/Checking + Symmetry + Contamination + g + age + gender + race + QualityMetric. SCS = Superior Corticostriatial, PTR = Posterior Thalamic Radiation, OFC = Orbitofrontal Cortex

| **Model: <ROI> <DV> ~ P-Factor x Age + Covariates** | *B* | *p_orig_* | *p_FDR_* |
| --- | --- | --- | --- |
| ROI: Cingulum Left |  |  |  |
| FC | 0.036 | 0.211 | 0.590 |
| FDC | 0.003 | 0.913 | 0.967 |
| FD | -0.050 | 0.094 | 0.486 |
| ROI: Cingulum Right |  |  |  |
| FC | 0.040 | 0.172 | 0.589 |
| FDC | 0.013 | 0.651 | 0.870 |
| FD | -0.036 | 0.230 | 0.605 |
| ROI: Callosum Body |  |  |  |
| FC | 0.024 | 0.422 | 0.709 |
| FDC | 0.027 | 0.346 | 0.644 |
| FD | 0.009 | 0.767 | 0.921 |
| ROI: Callosum Genu |  |  |  |
| FC | 0.036 | 0.290 | 0.609 |
| FDC | 0.012 | 0.704 | 0.870 |
| FD | -0.049 | 0.091 | 0.486 |
| ROI: Callosum Splenium |  |  |  |
| FC | -0.003 | 0.923 | 0.967 |
| FDC | -0.002 | 0.944 | 0.967 |
| FD | -0.034 | 0.290 | 0.609 |
| ROI: OFC Left |  |  |  |
| FC | 0.073 | 0.020 | 0.414 |
| FDC | 0.028 | 0.368 | 0.644 |
| FD | -0.062 | 0.059 | 0.486 |
| ROI: OFC Right |  |  |  |
| FC | 0.049 | 0.110 | 0.486 |
| FDC | 0.013 | 0.678 | 0.870 |
| FD | -0.059 | 0.080 | 0.486 |
| ROI: PTR Left |  |  |  |
| FC | -0.028 | 0.363 | 0.644 |
| FDC | -0.061 | 0.040 | 0.422 |
| FD | -0.072 | 0.031 | 0.422 |
| ROI: PTR Right |  |  |  |
| FC | 0.001 | 0.975 | 0.975 |
| FDC | -0.043 | 0.158 | 0.589 |
| FD | -0.085 | 0.012 | 0.414 |
| ROI: Saggital Striatum Left |  |  |  |
| FC | 0.038 | 0.194 | 0.589 |
| FDC | 0.004 | 0.885 | 0.967 |
| FD | -0.038 | 0.253 | 0.605 |
| ROI: Saggital Striatum Right |  |  |  |
| FC | 0.022 | 0.460 | 0.720 |
| FDC | 0.016 | 0.601 | 0.870 |
| FD | -0.003 | 0.937 | 0.967 |
| ROI: Uncinate Left |  |  |  |
| FC | -0.022 | 0.463 | 0.720 |
| FDC | -0.035 | 0.259 | 0.605 |
| FC | -0.030 | 0.360 | 0.644 |
| ROI: Uncinate Right |  |  |  |
| FC | 0.046 | 0.116 | 0.486 |
| FDC | 0.019 | 0.526 | 0.789 |
| FC | -0.007 | 0.831 | 0.967 |
| ROI: SCS Left |  |  |  |
| FC | 0.013 | 0.629 | 0.870 |
| FDC | -0.010 | 0.705 | 0.870 |
| FC | -0.042 | 0.196 | 0.589 |

**Supplemental table 3f:** standardized betas and p-values for the models testing the association between P-Factor x Age interaction term and the 14 fixel ROIs. The full model with covariates can be represented as ROI ~ (P-factor x Age) + P-factor + Bad Thoughts + Repetition/Checking + Symmetry + Contamination + g + age + gender + race + QualityMetric. SCS = Superior Corticostriatial, PTR = Posterior Thalamic Radiation, OFC = Orbitofrontal Cortex

| **Model: <ROI> <DV> ~ Repetition/Checking x Age + Covariates** | *B* | *p_orig_* | *p_FDR_* |
| --- | --- | --- | --- |
| ROI: Cingulum Left |  |  |  |
| FC | -0.002 | 0.941 | 0.984 |
| FDC | -0.023 | 0.459 | 0.918 |
| FD | -0.043 | 0.168 | 0.722 |
| ROI: Cingulum Right |  |  |  |
| FC | -0.013 | 0.670 | 0.918 |
| FDC | -0.032 | 0.307 | 0.859 |
| FD | -0.042 | 0.189 | 0.722 |
| ROI: Callosum Body |  |  |  |
| FC | 0.003 | 0.923 | 0.984 |
| FDC | 0.001 | 0.961 | 0.984 |
| FD | 0.009 | 0.780 | 0.963 |
| ROI: Callosum Genu |  |  |  |
| FC | 0.024 | 0.498 | 0.918 |
| FDC | -0.001 | 0.985 | 0.985 |
| FD | -0.052 | 0.086 | 0.722 |
| ROI: Callosum Splenium |  |  |  |
| FC | -0.023 | 0.482 | 0.918 |
| FDC | -0.016 | 0.616 | 0.918 |
| FD | -0.008 | 0.814 | 0.977 |
| ROI: OFC Left |  |  |  |
| FC | 0.020 | 0.547 | 0.918 |
| FDC | -0.020 | 0.547 | 0.918 |
| FD | -0.059 | 0.089 | 0.722 |
| ROI: OFC Right |  |  |  |
| FC | 0.027 | 0.400 | 0.918 |
| FDC | -0.022 | 0.500 | 0.918 |
| FD | -0.076 | 0.033 | 0.722 |
| ROI: PTR Left |  |  |  |
| FC | -0.039 | 0.234 | 0.755 |
| FDC | -0.058 | 0.068 | 0.722 |
| FD | -0.050 | 0.161 | 0.722 |
| ROI: PTR Right |  |  |  |
| FC | -0.034 | 0.296 | 0.859 |
| FDC | -0.044 | 0.165 | 0.722 |
| FD | -0.029 | 0.409 | 0.918 |
| ROI: Saggital Striatum Left |  |  |  |
| FC | -0.016 | 0.617 | 0.918 |
| FDC | -0.041 | 0.189 | 0.722 |
| FD | -0.034 | 0.330 | 0.865 |
| ROI: Saggital Striatum Right |  |  |  |
| FC | -0.013 | 0.680 | 0.918 |
| FDC | -0.011 | 0.717 | 0.918 |
| FD | 0.003 | 0.930 | 0.984 |
| ROI: Uncinate Left |  |  |  |
| FC | -0.011 | 0.721 | 0.918 |
| FDC | -0.003 | 0.929 | 0.984 |
| FC | 0.007 | 0.838 | 0.978 |
| ROI: Uncinate Right |  |  |  |
| FC | 0.048 | 0.119 | 0.722 |
| FDC | 0.048 | 0.134 | 0.722 |
| FC | 0.041 | 0.229 | 0.755 |
| ROI: SCS Left |  |  |  |
| FC | -0.018 | 0.520 | 0.918 |
| FDC | -0.013 | 0.656 | 0.918 |
| FC | 0.015 | 0.660 | 0.918 |

**Supplemental table 3g:** standardized betas and p-values for the models testing the association between the Repetition/Checking x Age interaction term and the 14 fixel ROIs. The full model with covariates can be represented as ROI ~ (Repetition/Checking x Age) + P-factor + Bad Thoughts + Repetition/Checking + Symmetry + Contamination + g + age + gender + race + QualityMetric. SCS = Superior Corticostriatial, PTR = Posterior Thalamic Radiation, OFC = Orbitofrontal Cortex

| **Model: <ROI> <DV> ~ Repetition/Checking x Age + Covariates** | *B* | *p_orig_* | *p_FDR_* |
| --- | --- | --- | --- |
| ROI: Cingulum Left |  |  |  |
| FC | -0.002 | 0.941 | 0.984 |
| FDC | -0.023 | 0.459 | 0.918 |
| FD | -0.043 | 0.168 | 0.722 |
| ROI: Cingulum Right |  |  |  |
| FC | -0.013 | 0.670 | 0.918 |
| FDC | -0.032 | 0.307 | 0.859 |
| FD | -0.042 | 0.189 | 0.722 |
| ROI: Callosum Body |  |  |  |
| FC | 0.003 | 0.923 | 0.984 |
| FDC | 0.001 | 0.961 | 0.984 |
| FD | 0.009 | 0.780 | 0.963 |
| ROI: Callosum Genu |  |  |  |
| FC | 0.024 | 0.498 | 0.918 |
| FDC | -0.001 | 0.985 | 0.985 |
| FD | -0.052 | 0.086 | 0.722 |
| ROI: Callosum Splenium |  |  |  |
| FC | -0.023 | 0.482 | 0.918 |
| FDC | -0.016 | 0.616 | 0.918 |
| FD | -0.008 | 0.814 | 0.977 |
| ROI: OFC Left |  |  |  |
| FC | 0.020 | 0.547 | 0.918 |
| FDC | -0.020 | 0.547 | 0.918 |
| FD | -0.059 | 0.089 | 0.722 |
| ROI: OFC Right |  |  |  |
| FC | 0.027 | 0.400 | 0.918 |
| FDC | -0.022 | 0.500 | 0.918 |
| FD | -0.076 | 0.033 | 0.722 |
| ROI: PTR Left |  |  |  |
| FC | -0.039 | 0.234 | 0.755 |
| FDC | -0.058 | 0.068 | 0.722 |
| FD | -0.050 | 0.161 | 0.722 |
| ROI: PTR Right |  |  |  |
| FC | -0.034 | 0.296 | 0.859 |
| FDC | -0.044 | 0.165 | 0.722 |
| FD | -0.029 | 0.409 | 0.918 |
| ROI: Saggital Striatum Left |  |  |  |
| FC | -0.016 | 0.617 | 0.918 |
| FDC | -0.041 | 0.189 | 0.722 |
| FD | -0.034 | 0.330 | 0.865 |
| ROI: Saggital Striatum Right |  |  |  |
| FC | -0.013 | 0.680 | 0.918 |
| FDC | -0.011 | 0.717 | 0.918 |
| FD | 0.003 | 0.930 | 0.984 |
| ROI: Uncinate Left |  |  |  |
| FC | -0.011 | 0.721 | 0.918 |
| FDC | -0.003 | 0.929 | 0.984 |
| FC | 0.007 | 0.838 | 0.978 |
| ROI: Uncinate Right |  |  |  |
| FC | 0.048 | 0.119 | 0.722 |
| FDC | 0.048 | 0.134 | 0.722 |
| FC | 0.041 | 0.229 | 0.755 |
| ROI: SCS Left |  |  |  |
| FC | -0.018 | 0.520 | 0.918 |
| FDC | -0.013 | 0.656 | 0.918 |
| FC | 0.015 | 0.660 | 0.918 |

**Supplemental table 3h:** standardized betas and p-values for the models testing the association between the Bad Thoughts x Age interaction term and the 14 fixel ROIs. The full model with covariates can be represented as ROI ~ (Bad Thoughts x Age) + P-factor + Bad Thoughts + Repetition/Checking + Symmetry + Contamination + g + age + gender + race + QualityMetric. SCS = Superior Corticostriatial, PTR = Posterior Thalamic Radiation, OFC = Orbitofrontal Cortex

| **Model: <ROI> <DV> ~ Symmetry x Age + Covariates** | *B* | *p_orig_* | *p_FDR_* |
| --- | --- | --- | --- |
| ROI: Cingulum Left |  |  |  |
| FC | 0.030 | 0.302 | 0.900 |
| FDC | -0.006 | 0.844 | 0.984 |
| FD | -0.054 | 0.074 | 0.772 |
| ROI: Cingulum Right |  |  |  |
| FC | 0.034 | 0.260 | 0.881 |
| FDC | -0.010 | 0.736 | 0.984 |
| FD | -0.065 | 0.033 | 0.472 |
| ROI: Callosum Body |  |  |  |
| FC | -0.006 | 0.832 | 0.984 |
| FDC | 0.018 | 0.533 | 0.984 |
| FD | 0.050 | 0.116 | 0.881 |
| ROI: Callosum Genu |  |  |  |
| FC | 0.030 | 0.376 | 0.900 |
| FDC | 0.031 | 0.356 | 0.900 |
| FD | -0.001 | 0.986 | 0.997 |
| ROI: Callosum Splenium |  |  |  |
| FC | -0.008 | 0.809 | 0.984 |
| FDC | 0.000 | 0.991 | 0.997 |
| FD | -0.006 | 0.867 | 0.984 |
| ROI: OFC Left |  |  |  |
| FC | 0.036 | 0.266 | 0.881 |
| FDC | -0.014 | 0.645 | 0.984 |
| FD | -0.071 | 0.034 | 0.472 |
| ROI: OFC Right |  |  |  |
| FC | 0.019 | 0.539 | 0.984 |
| FDC | -0.035 | 0.273 | 0.881 |
| FD | -0.089 | 0.009 | 0.389 |
| ROI: PTR Left |  |  |  |
| FC | -0.025 | 0.430 | 0.903 |
| FDC | -0.020 | 0.504 | 0.984 |
| FD | -0.004 | 0.912 | 0.997 |
| ROI: PTR Right |  |  |  |
| FC | -0.008 | 0.796 | 0.984 |
| FDC | -0.008 | 0.801 | 0.984 |
| FD | -0.003 | 0.934 | 0.997 |
| ROI: Saggital Striatum Left |  |  |  |
| FC | 0.027 | 0.359 | 0.900 |
| FDC | -0.014 | 0.638 | 0.984 |
| FD | -0.046 | 0.179 | 0.881 |
| ROI: Saggital Striatum Right |  |  |  |
| FC | 0.013 | 0.672 | 0.984 |
| FDC | -0.009 | 0.764 | 0.984 |
| FD | -0.030 | 0.394 | 0.900 |
| ROI: Uncinate Left |  |  |  |
| FC | -0.008 | 0.780 | 0.984 |
| FDC | -0.036 | 0.248 | 0.881 |
| FC | -0.044 | 0.190 | 0.881 |
| ROI: Uncinate Right |  |  |  |
| FC | 0.041 | 0.167 | 0.881 |
| FDC | -0.009 | 0.779 | 0.984 |
| FC | -0.039 | 0.238 | 0.881 |
| ROI: SCS Left |  |  |  |
| FC | 0.000 | 0.997 | 0.997 |
| FDC | -0.015 | 0.574 | 0.984 |
| FC | -0.027 | 0.407 | 0.900 |

**Supplemental table 3i:** standardized betas and p-values for the models testing the association between the Symmetry x Age interaction term and the 14 fixel ROIs. The full model with covariates can be represented as ROI ~ (Symmetry x Age) + P-factor + Bad Thoughts + Repetition/Checking + Symmetry + Contamination + g + age + gender + race + QualityMetric. SCS = Superior Corticostriatial, PTR = Posterior Thalamic Radiation, OFC = Orbitofrontal Cortex

| **Model: <ROI> <DV> ~ Contamination x Age + Covariates** | *B* | *p_orig_* | *p_FDR_* |
| --- | --- | --- | --- |
| ROI: Cingulum Left |  |  |  |
| FC | -0.031 | 0.282 | 0.839 |
| FDC | -0.048 | 0.102 | 0.742 |
| FD | -0.053 | 0.079 | 0.742 |
| ROI: Cingulum Right |  |  |  |
| FC | -0.029 | 0.320 | 0.839 |
| FDC | -0.040 | 0.186 | 0.839 |
| FD | -0.034 | 0.270 | 0.839 |
| ROI: Callosum Body |  |  |  |
| FC | 0.020 | 0.514 | 0.863 |
| FDC | 0.015 | 0.594 | 0.873 |
| FD | 0.003 | 0.935 | 0.957 |
| ROI: Callosum Genu |  |  |  |
| FC | 0.023 | 0.497 | 0.863 |
| FDC | 0.006 | 0.865 | 0.932 |
| FD | -0.047 | 0.106 | 0.742 |
| ROI: Callosum Splenium |  |  |  |
| FC | -0.025 | 0.428 | 0.863 |
| FDC | -0.023 | 0.461 | 0.863 |
| FD | -0.017 | 0.603 | 0.873 |
| ROI: OFC Left |  |  |  |
| FC | 0.006 | 0.838 | 0.926 |
| FDC | -0.019 | 0.547 | 0.873 |
| FD | -0.044 | 0.187 | 0.839 |
| ROI: OFC Right |  |  |  |
| FC | 0.012 | 0.696 | 0.926 |
| FDC | -0.021 | 0.497 | 0.863 |
| FD | -0.063 | 0.066 | 0.742 |
| ROI: PTR Left |  |  |  |
| FC | -0.061 | 0.048 | 0.742 |
| FDC | -0.060 | 0.046 | 0.742 |
| FD | -0.026 | 0.448 | 0.863 |
| ROI: PTR Right |  |  |  |
| FC | -0.034 | 0.274 | 0.839 |
| FDC | -0.040 | 0.190 | 0.839 |
| FD | -0.022 | 0.514 | 0.863 |
| ROI: Saggital Striatum Left |  |  |  |
| FC | -0.014 | 0.644 | 0.902 |
| FDC | -0.022 | 0.473 | 0.863 |
| FD | -0.009 | 0.786 | 0.926 |
| ROI: Saggital Striatum Right |  |  |  |
| FC | -0.011 | 0.718 | 0.926 |
| FDC | -0.009 | 0.752 | 0.926 |
| FD | 0.002 | 0.957 | 0.957 |
| ROI: Uncinate Left |  |  |  |
| FC | -0.031 | 0.301 | 0.839 |
| FDC | -0.036 | 0.242 | 0.839 |
| FC | -0.030 | 0.363 | 0.863 |
| ROI: Uncinate Right |  |  |  |
| FC | 0.033 | 0.268 | 0.839 |
| FDC | 0.018 | 0.562 | 0.873 |
| FC | 0.003 | 0.917 | 0.957 |
| ROI: SCS Left |  |  |  |
| FC | -0.006 | 0.812 | 0.926 |
| FDC | -0.007 | 0.789 | 0.926 |
| FC | 0.007 | 0.820 | 0.926 |

**Supplemental table 3j:** standardized betas and p-values for the models testing the association between the Contamination x Age interaction term and the 14 fixel ROIs. The full model with covariates can be represented as ROI ~ (Contamination x Age) + P-factor + Bad Thoughts + Repetition/Checking + Symmetry + Contamination + g + age + gender + race + QualityMetric. SCS = Superior Corticostriatial, PTR = Posterior Thalamic Radiation, OFC = Orbitofrontal Cortex

| **Model: <ROI> <DV> ~ OCS-factor + Covariates** | *B* | *p_orig_* | *p_FDR_* |
| --- | --- | --- | --- |
| ROI: Cingulum Left |  |  |  |
| FC | -0.020 | 0.597 | 0.993 |
| FDC | -0.016 | 0.671 | 0.993 |
| FD | -0.004 | 0.921 | 0.993 |
| ROI: Cingulum Right | 0.001 | 0.971 | 0.993 |
| FC | -0.013 | 0.745 | 0.993 |
| FDC | -0.032 | 0.418 | 0.993 |
| FD | -0.002 | 0.952 | 0.993 |
| ROI: Callosum Body | -0.030 | 0.425 | 0.993 |
| FC | -0.059 | 0.150 | 0.993 |
| FDC | -0.022 | 0.616 | 0.993 |
| FD | -0.027 | 0.524 | 0.993 |
| ROI: Callosum Genu | -0.024 | 0.534 | 0.993 |
| FC | 0.045 | 0.274 | 0.993 |
| FDC | 0.025 | 0.527 | 0.993 |
| FD | -0.040 | 0.351 | 0.993 |
| ROI: Callosum Splenium | 0.017 | 0.684 | 0.993 |
| FC | 0.007 | 0.870 | 0.993 |
| FDC | -0.009 | 0.843 | 0.993 |
| FD | 0.000 | 0.993 | 0.993 |
| ROI: OFC Left | -0.004 | 0.925 | 0.993 |
| FC | 0.004 | 0.933 | 0.993 |
| FDC | 0.031 | 0.448 | 0.993 |
| FD | -0.002 | 0.956 | 0.993 |
| ROI: OFC Right | -0.037 | 0.406 | 0.993 |
| FC | 0.047 | 0.242 | 0.993 |
| FDC | 0.047 | 0.237 | 0.993 |
| FD | 0.010 | 0.817 | 0.993 |
| ROI: PTR Left | -0.022 | 0.573 | 0.993 |
| FC | 0.003 | 0.930 | 0.993 |
| FDC | 0.033 | 0.447 | 0.993 |
| FD | 0.005 | 0.906 | 0.993 |
| ROI: PTR Right | -0.023 | 0.560 | 0.993 |
| FC | -0.042 | 0.355 | 0.993 |
| FDC | 0.006 | 0.882 | 0.993 |
| FD | 0.007 | 0.869 | 0.993 |
| ROI: Saggital Striatum Left | 0.013 | 0.773 | 0.993 |
| FC | -0.007 | 0.852 | 0.993 |
| FDC | -0.070 | 0.077 | 0.993 |
| FD | -0.099 | 0.021 | 0.863 |
| ROI: Saggital Striatum Right | 0.033 | 0.357 | 0.993 |
| FC | 0.016 | 0.646 | 0.993 |
| FDC | -0.027 | 0.529 | 0.993 |
| FD | -0.020 | 0.597 | 0.993 |
| ROI: Uncinate Left |  |  |  |
| FC |  |  |  |
| FDC |  |  |  |
| FC |  |  |  |
| ROI: Uncinate Right |  |  |  |
| FC |  |  |  |
| FDC |  |  |  |
| FC |  |  |  |
| ROI: SCS Left |  |  |  |
| FC |  |  |  |
| FDC |  |  |  |
| FC |  |  |  |

**Supplemental table 3k:** standardized betas and p-values for the models testing the association between the OCS-factor and the 14 fixel ROIs. The full model with covariates can be represented as ROI ~ OCS-factor + P-factor + Bad Thoughts + Repetition/Checking + Symmetry + Contamination + g + age + gender + race + QualityMetric. SCS = Superior Corticostriatial, PTR = Posterior Thalamic Radiation, OFC = Orbitofrontal Cortex

| **Model: <ROI> <DV> ~ OCS-factor x Age + Covariates** | *B* | *p_orig_* | *p_FDR_* |
| --- | --- | --- | --- |
| ROI: Cingulum Left |  |  |  |
| FC | -0.016 | 0.594 | 0.779 |
| FDC | -0.041 | 0.173 | 0.587 |
| FD | -0.061 | 0.047 | 0.435 |
| ROI: Cingulum Right |  |  |  |
| FC | -0.014 | 0.639 | 0.813 |
| FDC | -0.043 | 0.161 | 0.587 |
| FD | -0.058 | 0.063 | 0.443 |
| ROI: Callosum Body |  |  |  |
| FC | -0.003 | 0.917 | 0.940 |
| FDC | 0.010 | 0.734 | 0.856 |
| FD | 0.028 | 0.384 | 0.662 |
| ROI: Callosum Genu |  |  |  |
| FC | 0.014 | 0.680 | 0.840 |
| FDC | 0.006 | 0.853 | 0.914 |
| FD | -0.028 | 0.347 | 0.662 |
| ROI: Callosum Splenium |  |  |  |
| FC | -0.042 | 0.196 | 0.587 |
| FDC | -0.032 | 0.308 | 0.662 |
| FD | -0.009 | 0.796 | 0.880 |
| ROI: OFC Left |  |  |  |
| FC | 0.005 | 0.871 | 0.914 |
| FDC | -0.036 | 0.258 | 0.653 |
| FD | -0.068 | 0.049 | 0.435 |
| ROI: OFC Right |  |  |  |
| FC | 0.002 | 0.953 | 0.953 |
| FDC | -0.037 | 0.254 | 0.653 |
| FD | -0.068 | 0.052 | 0.435 |
| ROI: PTR Left |  |  |  |
| FC | -0.066 | 0.037 | 0.435 |
| FDC | -0.067 | 0.032 | 0.435 |
| FD | -0.031 | 0.377 | 0.662 |
| ROI: PTR Right |  |  |  |
| FC | -0.042 | 0.193 | 0.587 |
| FDC | -0.046 | 0.147 | 0.587 |
| FD | -0.021 | 0.547 | 0.741 |
| ROI: Saggital Striatum Left |  |  |  |
| FC | -0.021 | 0.499 | 0.698 |
| FDC | -0.054 | 0.080 | 0.479 |
| FD | -0.053 | 0.129 | 0.587 |
| ROI: Saggital Striatum Right |  |  |  |
| FC | -0.025 | 0.408 | 0.662 |
| FDC | -0.028 | 0.358 | 0.662 |
| FD | -0.013 | 0.724 | 0.856 |
| ROI: Uncinate Left |  |  |  |
| FC | -0.025 | 0.425 | 0.662 |
| FDC | -0.035 | 0.280 | 0.653 |
| FC | -0.028 | 0.413 | 0.662 |
| ROI: Uncinate Right |  |  |  |
| FC | 0.049 | 0.108 | 0.568 |
| FDC | 0.027 | 0.394 | 0.662 |
| FC | 0.010 | 0.775 | 0.880 |
| ROI: SCS Left |  |  |  |
| FC | -0.020 | 0.487 | 0.698 |
| FDC | -0.031 | 0.280 | 0.653 |
| FC | -0.025 | 0.459 | 0.688 |

**Supplemental table 3k:** standardized betas and p-values for the models testing the association between the OCS-factor x Age interaction term and the 14 fixel ROIs. The full model with covariates can be represented as ROI ~ (OCS-factor x Age) + P-factor + Bad Thoughts + Repetition/Checking + Symmetry + Contamination + g + age + gender + race + QualityMetric. SCS = Superior Corticostriatial, PTR = Posterior Thalamic Radiation, OFC = Orbitofrontal Cortex

**Tables S4a-l: Voxel-based (FA) ROI analyses**

| **Model: <ROI> <DV> ~ P-factor + Covariates** | *B* | *p_orig_* | *p_FDR_* |
| --- | --- | --- | --- |
| ROI: Cingulum Left FA | -0.032 | 0.416 | 0.762 |
| ROI: Cingulum Right FA | 0.020 | 0.613 | 0.792 |
| ROI: Callosum Body FA | 0.038 | 0.402 | 0.762 |
| ROI: Callosum Genu FA | 0.019 | 0.676 | 0.792 |
| ROI: Callosum Splenium FA | 0.011 | 0.808 | 0.870 |
| ROI: OFC Left FA | 0.018 | 0.679 | 0.792 |
| ROI: OFC Right FA | 0.026 | 0.552 | 0.792 |
| ROI: PTR Left FA | -0.104 | 0.025 | 0.343 |
| ROI: PTR Right FA | -0.089 | 0.058 | 0.405 |
| ROI: Saggital Striatum Left FA | -0.044 | 0.327 | 0.762 |
| ROI: Saggital Striatum Right FA | -0.035 | 0.434 | 0.762 |
| ROI: Uncinate Left FA | 0.005 | 0.912 | 0.912 |
| ROI: Uncinate Right FA | 0.067 | 0.140 | 0.652 |
| ROI: SCS Left FA | -0.035 | 0.435 | 0.762 |

**Supplemental table 4a:** standardized betas and p-values for the models testing the association between P-factor scores and the 14 voxel (FA) ROIs. The full model with covariates can be represented as ROI ~ P-factor + Bad Thoughts + Repetition/Checking + Symmetry + Contamination + g + age + gender + race + QualityMetric. SCS = Superior Corticostriatial, PTR = Posterior Thalamic Radiation, OFC = Orbitofrontal Cortex

| **Model: <ROI> <DV> ~ Repetition/Checking + Covariates** | *B* | *p_orig_* | *p_FDR_* |
| --- | --- | --- | --- |
| ROI: Cingulum Left FA | -0.019 | 0.647 | 0.697 |
| ROI: Cingulum Right FA | -0.046 | 0.259 | 0.453 |
| ROI: Callosum Body FA | -0.065 | 0.175 | 0.351 |
| ROI: Callosum Genu FA | -0.076 | 0.109 | 0.294 |
| ROI: Callosum Splenium FA | -0.074 | 0.126 | 0.294 |
| ROI: OFC Left FA | 0.014 | 0.758 | 0.758 |
| ROI: OFC Right FA | -0.040 | 0.383 | 0.536 |
| ROI: PTR Left FA | -0.029 | 0.548 | 0.639 |
| ROI: PTR Right FA | -0.046 | 0.352 | 0.536 |
| ROI: Saggital Striatum Left FA | -0.037 | 0.434 | 0.552 |
| ROI: Saggital Striatum Right FA | -0.101 | 0.034 | 0.159 |
| ROI: Uncinate Left FA | -0.109 | 0.023 | 0.159 |
| ROI: Uncinate Right FA | -0.121 | 0.012 | 0.159 |
| ROI: SCS Left FA | -0.075 | 0.112 | 0.294 |

**Supplemental table 4b:** standardized betas and p-values for the models testing the association between Repetition/Checking scores and the 14 voxel (FA) ROIs. The full model with covariates can be represented as ROI ~ P-factor + Bad Thoughts + Repetition/Checking + Symmetry + Contamination + g + age + gender + race + QualityMetric. SCS = Superior Corticostriatial, PTR = Posterior Thalamic Radiation, OFC = Orbitofrontal Cortex

| **Model: <ROI> <DV> ~ Bad Thoughts + Covariates** | *B* | *p_orig_* | *p_FDR_* |
| --- | --- | --- | --- |
| ROI: Cingulum Left FA | 0.054 | 0.172 | 0.372 |
| ROI: Cingulum Right FA | 0.032 | 0.407 | 0.519 |
| ROI: Callosum Body FA | 0.072 | 0.115 | 0.322 |
| ROI: Callosum Genu FA | 0.074 | 0.104 | 0.322 |
| ROI: Callosum Splenium FA | 0.054 | 0.239 | 0.372 |
| ROI: OFC Left FA | 0.056 | 0.202 | 0.372 |
| ROI: OFC Right FA | 0.041 | 0.350 | 0.491 |
| ROI: PTR Left FA | 0.100 | 0.030 | 0.168 |
| ROI: PTR Right FA | 0.098 | 0.036 | 0.168 |
| ROI: Saggital Striatum Left FA | 0.002 | 0.960 | 0.960 |
| ROI: Saggital Striatum Right FA | 0.032 | 0.484 | 0.539 |
| ROI: Uncinate Left FA | 0.031 | 0.501 | 0.539 |
| ROI: Uncinate Right FA | -0.055 | 0.225 | 0.372 |
| ROI: SCS Left FA | 0.110 | 0.014 | 0.168 |

**Supplemental table 4c:** standardized betas and p-values for the models testing the association between Bad Thoughts scores and the 14 voxel (FA) ROIs. The full model with covariates can be represented as ROI ~ P-factor + Bad Thoughts + Repetition/Checking + Symmetry + Contamination + g + age + gender + race + QualityMetric. SCS = Superior Corticostriatial, PTR = Posterior Thalamic Radiation, OFC = Orbitofrontal Cortex

| **Model: <ROI> <DV> ~ Symmetry + Covariates** | *B* | *p_orig_* | *p_FDR_* |
| --- | --- | --- | --- |
| ROI: Cingulum Left FA | -0.054 | 0.153 | 0.388 |
| ROI: Cingulum Right FA | -0.016 | 0.662 | 0.713 |
| ROI: Callosum Body FA | -0.042 | 0.339 | 0.527 |
| ROI: Callosum Genu FA | -0.065 | 0.136 | 0.388 |
| ROI: Callosum Splenium FA | -0.086 | 0.049 | 0.388 |
| ROI: OFC Left FA | -0.062 | 0.146 | 0.388 |
| ROI: OFC Right FA | -0.048 | 0.261 | 0.456 |
| ROI: PTR Left FA | -0.034 | 0.443 | 0.563 |
| ROI: PTR Right FA | -0.028 | 0.528 | 0.616 |
| ROI: Saggital Striatum Left FA | -0.073 | 0.089 | 0.388 |
| ROI: Saggital Striatum Right FA | -0.059 | 0.176 | 0.388 |
| ROI: Uncinate Left FA | -0.057 | 0.194 | 0.388 |
| ROI: Uncinate Right FA | 0.005 | 0.913 | 0.913 |
| ROI: SCS Left FA | -0.034 | 0.433 | 0.563 |

**Supplemental table 4d:** standardized betas and p-values for the models testing the association between Symmetry scores and the 14 voxel (FA) ROIs. The full model with covariates can be represented as ROI ~ P-factor + Bad Thoughts + Repetition/Checking + Symmetry + Contamination + g + age + gender + race + QualityMetric. SCS = Superior Corticostriatial, PTR = Posterior Thalamic Radiation, OFC = Orbitofrontal Cortex

| **Model: <ROI> <DV> ~ Contamination + Covariates** | *B* | *p_orig_* | *p_FDR_* |
| --- | --- | --- | --- |
| ROI: Cingulum Left FA | 0.019 | 0.622 | 0.825 |
| ROI: Cingulum Right FA | -0.011 | 0.766 | 0.825 |
| ROI: Callosum Body FA | -0.017 | 0.693 | 0.825 |
| ROI: Callosum Genu FA | -0.025 | 0.573 | 0.825 |
| ROI: Callosum Splenium FA | 0.057 | 0.199 | 0.696 |
| ROI: OFC Left FA | -0.024 | 0.583 | 0.825 |
| ROI: OFC Right FA | 0.017 | 0.700 | 0.825 |
| ROI: PTR Left FA | -0.005 | 0.919 | 0.919 |
| ROI: PTR Right FA | -0.016 | 0.729 | 0.825 |
| ROI: Saggital Striatum Left FA | 0.101 | 0.020 | 0.284 |
| ROI: Saggital Striatum Right FA | 0.073 | 0.095 | 0.578 |
| ROI: Uncinate Left FA | 0.048 | 0.276 | 0.773 |
| ROI: Uncinate Right FA | 0.068 | 0.124 | 0.578 |
| ROI: SCS Left FA | 0.030 | 0.486 | 0.825 |

**Supplemental table 4e:** standardized betas and p-values for the models testing the association between Contamination scores and the 14 voxel (FA) ROIs. The full model with covariates can be represented as ROI ~ P-factor + Bad Thoughts + Repetition/Checking + Symmetry + Contamination + g + age + gender + race + QualityMetric. SCS = Superior Corticostriatial, PTR = Posterior Thalamic Radiation, OFC = Orbitofrontal Cortex

| **Model: <ROI> <DV> ~ P-Factor x Age + Covariates** | *B* | *p_orig_* | *p_FDR_* |
| --- | --- | --- | --- |
| ROI: Cingulum Left FA | -0.045 | 0.117 | 0.234 |
| ROI: Cingulum Right FA | -0.046 | 0.112 | 0.234 |
| ROI: Callosum Body FA | 0.003 | 0.918 | 0.918 |
| ROI: Callosum Genu FA | -0.034 | 0.304 | 0.472 |
| ROI: Callosum Splenium FA | -0.005 | 0.892 | 0.918 |
| ROI: OFC Left FA | -0.076 | 0.019 | 0.133 |
| ROI: OFC Right FA | -0.078 | 0.017 | 0.133 |
| ROI: PTR Left FA | -0.062 | 0.069 | 0.234 |
| ROI: PTR Right FA | -0.060 | 0.083 | 0.234 |
| ROI: Saggital Striatum Left FA | -0.046 | 0.165 | 0.289 |
| ROI: Saggital Striatum Right FA | -0.054 | 0.108 | 0.234 |
| ROI: Uncinate Left FA | -0.010 | 0.755 | 0.918 |
| ROI: Uncinate Right FA | -0.004 | 0.910 | 0.918 |
| ROI: SCS Left FA | -0.017 | 0.613 | 0.858 |

**Supplemental table 4f:** standardized betas and p-values for the models testing the association between the P-factor x Age interaction term and the 14 voxel (FA) ROIs. The full model with covariates can be represented as ROI ~ (P-factor x Age) + P-factor + Bad Thoughts + Repetition/Checking + Symmetry + Contamination + g + age + gender + race + QualityMetric. SCS = Superior Corticostriatial, PTR = Posterior Thalamic Radiation, OFC = Orbitofrontal Cortex

| **Model: <ROI> <DV> ~ Repetition/Checking x Age + Covariates** | *B* | *p_orig_* | *p_FDR_* |
| --- | --- | --- | --- |
| ROI: Cingulum Left FA | -0.055 | 0.070 | 0.328 |
| ROI: Cingulum Right FA | -0.031 | 0.316 | 0.491 |
| ROI: Callosum Body FA | 0.015 | 0.670 | 0.670 |
| ROI: Callosum Genu FA | -0.036 | 0.304 | 0.491 |
| ROI: Callosum Splenium FA | 0.022 | 0.539 | 0.581 |
| ROI: OFC Left FA | -0.036 | 0.297 | 0.491 |
| ROI: OFC Right FA | -0.068 | 0.046 | 0.328 |
| ROI: PTR Left FA | -0.028 | 0.443 | 0.516 |
| ROI: PTR Right FA | 0.032 | 0.381 | 0.491 |
| ROI: Saggital Striatum Left FA | -0.033 | 0.346 | 0.491 |
| ROI: Saggital Striatum Right FA | -0.031 | 0.386 | 0.491 |
| ROI: Uncinate Left FA | 0.034 | 0.339 | 0.491 |
| ROI: Uncinate Right FA | 0.068 | 0.057 | 0.328 |
| ROI: SCS Left FA | 0.033 | 0.341 | 0.491 |

**Supplemental table 4g:** standardized betas and p-values for the models testing the association between the Repetition/Checking x Age interaction term and the 14 voxel (FA) ROIs. The full model with covariates can be represented as ROI ~ (Repetition/Checking x Age) + P-factor + Bad Thoughts + Repetition/Checking + Symmetry + Contamination + g + age + gender + race + QualityMetric. SCS = Superior Corticostriatial, PTR = Posterior Thalamic Radiation, OFC = Orbitofrontal Cortex

| **Model: <ROI> <DV> ~ Bad Thoughts x Age + Covariates** | *B* | *p_orig_* | *p_FDR_* |
| --- | --- | --- | --- |
| ROI: Cingulum Left FA | -0.038 | 0.216 | 0.755 |
| ROI: Cingulum Right FA | -0.038 | 0.215 | 0.755 |
| ROI: Callosum Body FA | 0.018 | 0.612 | 0.880 |
| ROI: Callosum Genu FA | -0.009 | 0.789 | 0.880 |
| ROI: Callosum Splenium FA | -0.012 | 0.733 | 0.880 |
| ROI: OFC Left FA | -0.033 | 0.334 | 0.850 |
| ROI: OFC Right FA | -0.031 | 0.364 | 0.850 |
| ROI: PTR Left FA | -0.010 | 0.775 | 0.880 |
| ROI: PTR Right FA | 0.006 | 0.861 | 0.880 |
| ROI: Saggital Striatum Left FA | -0.066 | 0.058 | 0.755 |
| ROI: Saggital Striatum Right FA | -0.044 | 0.215 | 0.755 |
| ROI: Uncinate Left FA | -0.024 | 0.497 | 0.880 |
| ROI: Uncinate Right FA | -0.012 | 0.738 | 0.880 |
| ROI: SCS Left FA | 0.005 | 0.880 | 0.880 |

**Supplemental table 4h:** standardized betas and p-values for the models testing the association between the Bad Thoughts x Age interaction term and the 14 voxel (FA) ROIs. The full model with covariates can be represented as ROI ~ (Bad Thoughts x Age) + P-factor + Bad Thoughts + Repetition/Checking + Symmetry + Contamination + g + age + gender + race + QualityMetric. SCS = Superior Corticostriatial, PTR = Posterior Thalamic Radiation, OFC = Orbitofrontal Cortex

| **Model: <ROI> <DV> ~ Symmetry x Age + Covariates** | *B* | *p_orig_* | *p_FDR_* |
| --- | --- | --- | --- |
| ROI: Cingulum Left FA | -0.036 | 0.224 | 0.392 |
| ROI: Cingulum Right FA | -0.041 | 0.163 | 0.366 |
| ROI: Callosum Body FA | 0.063 | 0.066 | 0.307 |
| ROI: Callosum Genu FA | -0.005 | 0.882 | 0.949 |
| ROI: Callosum Splenium FA | 0.046 | 0.179 | 0.366 |
| ROI: OFC Left FA | -0.065 | 0.048 | 0.307 |
| ROI: OFC Right FA | -0.093 | 0.005 | 0.070 |
| ROI: PTR Left FA | -0.012 | 0.735 | 0.857 |
| ROI: PTR Right FA | 0.019 | 0.584 | 0.743 |
| ROI: Saggital Striatum Left FA | -0.045 | 0.183 | 0.366 |
| ROI: Saggital Striatum Right FA | -0.048 | 0.161 | 0.366 |
| ROI: Uncinate Left FA | -0.029 | 0.399 | 0.620 |
| ROI: Uncinate Right FA | 0.001 | 0.982 | 0.982 |
| ROI: SCS Left FA | 0.022 | 0.518 | 0.726 |

**Supplemental table 4i:** standardized betas and p-values for the models testing the association between the Symmetry x Age interaction term and the 14 voxel (FA) ROIs. The full model with covariates can be represented as ROI ~ (Symmetry x Age) + P-factor + Bad Thoughts + Repetition/Checking + Symmetry + Contamination + g + age + gender + race + QualityMetric. SCS = Superior Corticostriatial, PTR = Posterior Thalamic Radiation, OFC = Orbitofrontal Cortex

| **Model: <ROI> <DV> ~ Contamination x Age + Covariates** | *B* | *p_orig_* | *p_FDR_* |
| --- | --- | --- | --- |
| ROI: Cingulum Left FA | -0.047 | 0.109 | 0.743 |
| ROI: Cingulum Right FA | -0.030 | 0.303 | 0.743 |
| ROI: Callosum Body FA | 0.010 | 0.771 | 0.956 |
| ROI: Callosum Genu FA | -0.007 | 0.824 | 0.956 |
| ROI: Callosum Splenium FA | -0.004 | 0.916 | 0.956 |
| ROI: OFC Left FA | -0.033 | 0.318 | 0.743 |
| ROI: OFC Right FA | -0.037 | 0.252 | 0.743 |
| ROI: PTR Left FA | -0.007 | 0.838 | 0.956 |
| ROI: PTR Right FA | 0.019 | 0.588 | 0.956 |
| ROI: Saggital Striatum Left FA | -0.029 | 0.386 | 0.772 |
| ROI: Saggital Striatum Right FA | -0.040 | 0.232 | 0.743 |
| ROI: Uncinate Left FA | 0.002 | 0.956 | 0.956 |
| ROI: Uncinate Right FA | 0.009 | 0.785 | 0.956 |
| ROI: SCS Left FA | 0.037 | 0.267 | 0.743 |

**Supplemental table 4j:** standardized betas and p-values for the models testing the association between the Contamination x Age interaction term and the 14 voxel (FA) ROIs. The full model with covariates can be represented as ROI ~ (Contamination x Age) + P-factor + Bad Thoughts + Repetition/Checking + Symmetry + Contamination + g + age + gender + race + QualityMetric. SCS = Superior Corticostriatial, PTR = Posterior Thalamic Radiation, OFC = Orbitofrontal Cortex

| **Model: <ROI> <DV> ~ OCS Factor + Covariates** | *B* | *p_orig_* | *p_FDR_* |
| --- | --- | --- | --- |
| ROI: Cingulum Left FA | -0.052 | 0.085 | 0.396 |
| ROI: Cingulum Right FA | -0.035 | 0.239 | 0.558 |
| ROI: Callosum Body FA | 0.034 | 0.332 | 0.631 |
| ROI: Callosum Genu FA | -0.015 | 0.672 | 0.787 |
| ROI: Callosum Splenium FA | 0.011 | 0.765 | 0.824 |
| ROI: OFC Left FA | -0.049 | 0.149 | 0.522 |
| ROI: OFC Right FA | -0.064 | 0.060 | 0.396 |
| ROI: PTR Left FA | -0.015 | 0.674 | 0.787 |
| ROI: PTR Right FA | 0.027 | 0.451 | 0.631 |
| ROI: Saggital Striatum Left FA | -0.062 | 0.070 | 0.396 |
| ROI: Saggital Striatum Right FA | -0.045 | 0.194 | 0.543 |
| ROI: Uncinate Left FA | 0.000 | 0.995 | 0.995 |
| ROI: Uncinate Right FA | 0.027 | 0.436 | 0.631 |
| ROI: SCS Left FA | 0.031 | 0.371 | 0.631 |

**Supplemental table 4k:** standardized betas and p-values for the models testing the association between the OCS-factor and the 14 voxel (FA) ROIs. The full model with covariates can be represented as ROI ~ OCS-factor + P-factor + Bad Thoughts + Repetition/Checking + Symmetry + Contamination + g + age + gender + race + QualityMetric. SCS = Superior Corticostriatial, PTR = Posterior Thalamic Radiation, OFC = Orbitofrontal Cortex

Symmetry + Contamination + g + age + gender + race + QualityMetric. SCS = Superior Corticostriatial, PTR = Posterior Thalamic Radiation, OFC = Orbitofrontal Cortex

| **Model: <ROI> <DV> ~ OCS Factor x Age + Covariates** | *B* | *p_orig_* | *p_FDR_* |
| --- | --- | --- | --- |
| ROI: Cingulum Left FA | -0.006 | 0.871 | 0.938 |
| ROI: Cingulum Right FA | -0.040 | 0.289 | 0.578 |
| ROI: Callosum Body FA | -0.053 | 0.229 | 0.559 |
| ROI: Callosum Genu FA | -0.090 | 0.040 | 0.267 |
| ROI: Callosum Splenium FA | -0.052 | 0.239 | 0.559 |
| ROI: OFC Left FA | -0.015 | 0.720 | 0.917 |
| ROI: OFC Right FA | -0.033 | 0.445 | 0.779 |
| ROI: PTR Left FA | 0.019 | 0.680 | 0.917 |
| ROI: PTR Right FA | -0.002 | 0.963 | 0.963 |
| ROI: Saggital Striatum Left FA | -0.011 | 0.799 | 0.932 |
| ROI: Saggital Striatum Right FA | -0.059 | 0.182 | 0.559 |
| ROI: Uncinate Left FA | -0.084 | 0.057 | 0.267 |
| ROI: Uncinate Right FA | -0.092 | 0.039 | 0.267 |
| ROI: SCS Left FA | 0.017 | 0.693 | 0.917 |

**Supplemental table 4l:** standardized betas and p-values for the models testing the association between the OCS Factor x Age interaction term and the 14 voxel (FA) ROIs. The full model with covariates can be represented as ROI ~ (OCS Factor x Age) + P-factor + Bad Thoughts + Repetition/Checking + Symmetry + Contamination + g + age + gender + race + QualityMetric. SCS = Superior Corticostriatial, PTR = Posterior Thalamic Radiation, OFC = Orbitofrontal Cortex

**Table S5:** exploratory models for nonlinear age covariate and age-interaction effects

| **Model** | **FA** | **FD** | **FC** | **FDC** |
| --- | --- | --- | --- | --- |
| Whole Brain F(A/c/d/dc) = sex + age + age^2 + race + Qcmetric + g + **Pfactor** + OCDBadThoughts + OCDRepChk + OCDSymm + OCDContam | *ns* | *ns* | *ns* | *ns* |
| Whole Brain F(A/c/d/dc) = sex + age + age^2 + race + Qcmetric + g - **Pfactor** + OCDBadThoughts + OCDRepChk + OCDSymm + OCDContam | *** results are equivalent with or without squared age term** | *ns* | *ns* | *ns* |
| Whole Brain F(A/c/d/dc) = sex + age + age^2 + race + Qcmetric + g + Pfactor + **OCDBadThoughts** + OCDRepChk + OCDSymm + OCDContam | *ns* | *** results are equivalent with or without squared age term** | *ns* | *ns* |
| Whole Brain F(A/c/d/dc) = sex + age + age^2 + race + Qcmetric + g + Pfactor - **OCDBadThoughts** + OCDRepChk + OCDSymm + OCDContam | *ns* | *ns* | *ns* | *ns* |
| Whole Brain F(A/c/d/dc) = sex + age + age^2 + race + Qcmetric + g + Pfactor + OCDBadThoughts + **OCDRepChk** + OCDSymm + OCDContam | *ns* | *ns* | *** results are equivalent with or without squared age term** | *ns* |
| Whole Brain F(A/c/d/dc) = sex + age + age^2 + race + Qcmetric + g + Pfactor + OCDBadThoughts - **OCDRepChk** + OCDSymm + OCDContam | *** results are equivalent with or without squared age term** | *ns* | *ns* | *ns* |
| Whole Brain F(A/c/d/dc) = sex + age + age^2 + race + Qcmetric + g + Pfactor + OCDBadThoughts + OCDRepChk + **OCDSymm** + OCDContam | *ns* | *ns* | *ns* | *ns* |
| Whole Brain F(A/c/d/dc) = sex + age + age^2 + race + Qcmetric + g + Pfactor + OCDBadThoughts + OCDRepChk - **OCDSymm** + OCDContam | *ns* | *ns* | *ns* | *** results are equivalent with or without squared age term** |
| Whole Brain F(A/c/d/dc) = sex + age + age^2 + race + Qcmetric + g + Pfactor + OCDBadThoughts + OCDRepChk + OCDSymm + **OCDContam** | *ns* | *ns* | *ns* | *ns* |
| Whole Brain F(A/c/d/dc) = sex + age + age^2 + race + Qcmetric + g + Pfactor + OCDBadThoughts + OCDRepChk + OCDSymm - **OCDContam** | *ns* | *ns* | *ns* | *ns* |
| Whole Brain F(A/c/d/dc) = sex + age + age^2 + race + Qcmetric + g + Pfactor + OCDBadThoughts + OCDRepChk + OCDSymm + OCDContam + (Pfactor*age) + **(Pfactor*age^2)** | *ns* | *ns* | *ns* | *ns* |
| Whole Brain F(A/c/d/dc) = sex + age + age^2 + race + Qcmetric + g + Pfactor + OCDBadThoughts + OCDRepChk + OCDSymm + OCDContam + (Pfactor*age) - **(Pfactor*age^2)** | *ns [original contrast with linear age interaction was significant]* | *ns* | *ns* | *ns* |
| Whole Brain F(A/c/d/dc) = sex + age + age^2 + race + Qcmetric + g + Pfactor + OCDBadThoughts + OCDRepChk + OCDSymm + OCDContam + (OCDRepChk*age) + **(OCDRepChk*age^2)** | *ns* | *ns* | *ns* | *ns* |
| Whole Brain F(A/c/d/dc) = sex + age + age^2 + race + Qcmetric + g + Pfactor + OCDBadThoughts + OCDRepChk + OCDSymm + OCDContam + (OCDRepChk*age) - **(OCDRepChk*age^2)** | *ns* | *ns* | * **(NEW RESULT with squared age interaction term)** | *ns* |
| Whole Brain F(A/c/d/dc) = sex + age + age^2 + race + Qcmetric + g + Pfactor + OCDBadThoughts + OCDRepChk + OCDSymm + OCDContam + (OCDSymm*age) + **(OCDSymm*age^2)** | *ns* | *ns* | *ns* | *ns* |
| Whole Brain F(A/c/d/dc) = sex + age + age^2 + race + Qcmetric + g + Pfactor + OCDBadThoughts + OCDRepChk + OCDSymm + OCDContam + (OCDSymm*age) - **(OCDSymm*age^2)** | *ns* | *ns* | *ns* | *ns* |
| Whole Brain F(A/c/d/dc) = sex + age + age^2 + race + Qcmetric + g + Pfactor + OCDBadThoughts + OCDRepChk + OCDSymm + OCDContam + (OCDBadThoughts*age) + **(OCDBadThoughts*age^2)** | *ns* | *ns* | *ns* | *ns* |
| Whole Brain F(A/c/d/dc) = sex + age + age^2 + race + Qcmetric + g + Pfactor + OCDBadThoughts + OCDRepChk + OCDSymm + OCDContam + (OCDBadThoughts*age) - **(OCDBadThoughts*age^2)** | *ns* | *ns* | *ns* | *ns* |
| Whole Brain F(A/c/d/dc) = sex + age + age^2 + race + Qcmetric + g + Pfactor + OCDBadThoughts + OCDRepChk + OCDSymm + OCDContam + (OCDContam*age) + **(OCDContam*age^2)** | *ns* | *ns* | *ns* | *ns* |
| Whole Brain F(A/c/d/dc) = sex + age + age^2 + race + Qcmetric + g + Pfactor + OCDBadThoughts + OCDRepChk + OCDSymm + OCDContam + (OCDContam*age) - **(OCDContam*age^2)** | *ns* | *ns* | *ns* | *ns* |
| Whole Brain F(A/c/d/dc) = sex + age + age^2 + race + Qcmetric + g + Pfactor + OCDfactor + (OCDfactor*age) + **(OCDfactor*age^2)** | *ns* | *ns* | *ns* | *ns* |
| Whole Brain F(A/c/d/dc) = sex + age + age^2 + race + Qcmetric + g + Pfactor + OCDfactor + (OCDfactor*age) - **(OCDfactor*age^2)** | *ns* | *ns* | *ns* | *ns* |

**Table S6:** exploratory models for age effects

| **Model** | **FA** | **FD** | **FC** | **FDC** |
| --- | --- | --- | --- | --- |
| Whole Brain F(A/c/d/dc) = sex + **age** + race + Qcmetric | * | * | * | * |
| Whole Brain F(A/c/d/dc) = sex - **age** + race + Qcmetric | * | * | * | * |
| Whole Brain F(A/c/d/dc) = sex + age + **age^2** + race + Qcmetric | ns | ns | ns | ns |
| Whole Brain F(A/c/d/dc) = sex + age - **age^2** + race + Qcmetric | * | * | * | * |

| **Table S7:** exploratory models for *g*-factor (IQ) effects  **Model** | **FA** | **FD** | **FC** | **FDC** |
| --- | --- | --- | --- | --- |
| Whole Brain F(A/c/d/dc) = sex + age + race + Qcmetric + g + Pfactor **+ gIQ** | *ns* | *ns* | * | * |
| Whole Brain F(A/c/d/dc) = sex + age + race + Qcmetric + g + Pfactor - **gIQ** | *ns* | *ns* | *ns* | *ns* |
| Whole Brain F(A/c/d/dc) = sex + age + race + Qcmetric + g + Pfactor + gIQ + **(gIQ*age)** | *ns* | * | *ns* | *ns* |
| Whole Brain F(A/c/d/dc) = sex + age + race + Qcmetric + g + Pfactor + gIQ - **(gIQ*age)** | * | *ns* | *ns* | *ns* |
| Whole Brain F(A/c/d/dc) = sex + age + age^2 + race + Qcmetric + g + Pfactor + gIQ + (gIQ*age) + **(gIQ*age^2)** | *ns* | *ns* | *ns* | *ns* |
| Whole Brain F(A/c/d/dc) = sex + age + age^2 + race + Qcmetric + g + Pfactor + gIQ + (gIQ*age) - **(gIQ*age^2)** | *ns* | *ns* | *ns* | *ns* |
